# Global Change Asymmetrically Rewires Ecosystems

**DOI:** 10.1111/ele.70174

**Published:** 2025-07-10

**Authors:** Charlotte A. Ward, Tyler D. Tunney, Ian Donohue, Carling Bieg, Kayla R. S. Hale, Bailey C. McMeans, John C. Moore, Kevin S. McCann

**Affiliations:** ^1^ University of Guelph Guelph Ontario Canada; ^2^ Fisheries and Oceans Canada Moncton New Brunswick Canada; ^3^ Trinity College Dublin Dublin Ireland; ^4^ Case Western Reserve University Cleveland Ohio USA; ^5^ University of Toronto Mississauga Mississauga Toronto Canada; ^6^ Colorado State University Fort Collins Colorado USA

**Keywords:** asymmetry, ecosystem function, food web, global change, habitat coupling, resilience, rewiring

## Abstract

Global change is complex and multidimensional, making it challenging to understand how human activities affect ecosystem processes. A critical gap in this understanding is how drivers of global change broadly affect food webs. While an industry of studies documents shifts in food webs in response to anthropogenic pressures, a general synthesis is lacking. To address this, we review studies across diverse ecosystems that use stable isotope analysis, energetic food web modelling and gut content analysis to reveal the prevalence of asymmetric rewiring—a phenomenon whereby anthropogenic pressures differentially impact habitats across space, altering some energy pathways within food webs relative to others. We then highlight several examples from the literature to illustrate how this process unfolds. To explore its broader consequences, we use a simple food web model to demonstrate how asymmetric rewiring alters resilience and key ecosystem functions, such as primary and secondary production. Our synthesis uncovers a remarkably general response in food web structure to global change that needs to be better understood to protect nature and the services that human societies rely on in a rapidly changing world.

## Graphical abstract

Understanding how global environmental change affects food webs remains a key challenge. We synthesise empirical studies to reveal a general pattern of asymmetric rewiring, where anthropogenic pressures differentially impact habitats and restructure energy pathways within food webs. We show that asymmetric rewiring alters resilience and key ecosystem functions, highlighting a widespread yet underappreciated response of food webs to global change.

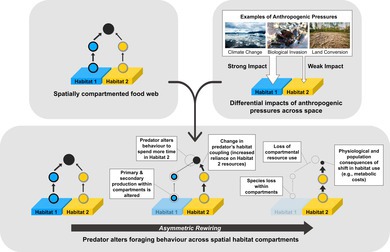

## 1 Introduction

The activities of 8 billion people on the planet are generating novel environmental pressures, causing a suite of human‐driven *global changes* (e.g., climate change, land conversion, biological invasions and pollution; Millennium Ecosystem Assessment 2005; Nelson et al. 2006; see definitions of italicised words in Table 1). Indeed, recent work has introduced the idea that climate change impacts distinct habitats differentially (Bartley et al. 2019; Tunney et al. 2014). *Generalist consumer* species then respond to these changes by shifting their habitat use (e.g., for foraging). These habitat changes and concomitant consumer responses ultimately alter the spatial structure of food webs and lead to the ‘rewiring’ of the topological patterning (who eats whom) and strength (how much) of consumptive interactions (Bartley et al. 2019; Blanchard 2015). In principle, however, this rewiring could arise from any form of *anthropogenic pressure*, whether acting at the scale of microhabitats or spanning whole ecosystems; yet this possibility has not yet been addressed in the ecological literature. Consequently, it is imperative that we better understand this novel source of rewiring in response to global change, hereafter termed *asymmetric rewiring*, to protect nature and the ecological services that human societies rely on.

**TABLE 1:** Definitions of key terms and concepts related to the asymmetric rewiring of food webs under global change.

Term	Definition
Asymmetry	The disproportion in the spatial arrangement of sides or parts of an entity (Merriam‐Webster 2024)
Global change	Broad‐scale changes to the Earth's abiotic and biotic environment caused by human activities, impacting climate, ecosystems and their processes, and biodiversity (Steffen et al. 2005)
Anthropogenic pressure	Forces exerted on the environment resulting from human activities that have the potential to degrade or disrupt natural systems (Danet et al. 2024; Venter et al. 2016)
Generalist consumer	Organisms, typically at higher trophic levels (e.g., secondary consumers and predators), that move and forage across multiple habitat types and rely on a variety of food sources (McCann et al. 2005)
Habitat coupling	The linkage of spatial sites characterised by distinct environmental conditions by the transmission of material, energy or nutrients. This transmission may be driven by physical processes or by the feeding of mobile generalist consumers (Dolson et al. 2009; McMeans et al. 2016)
Energy pathway	The transfer of energy through a sequence of feeding relationships from an organism to another within a food web. For example, from primary producers to consumers to top predators (Vadeboncoeur et al. 2002)
Trophospecies	A group of species that use the same resources and/or occupy the same trophic level (Embke and Vander Zanden 2022)
Asymmetric rewiring	A spatially disproportionate restructuring of food webs that may result from organismal responses to differential change in distinct habitats associated with natural or human disturbance
Interaction strength rewiring	A form of asymmetric rewiring that arises from changes in the magnitude of energy flow (e.g., biomass or carbon) from one species to another. This may result from shifts in consumer behaviour driven by changes in consumptive demand, or from mechanisms related to prey availability (Bartley et al. 2019). It also encompasses other definitions of interaction strength, such as disproportionate changes in the effect of one species on another (*sensu* Paine 1980)
Topological rewiring	A form of asymmetric rewiring that arises from changes in the presence or absence of interactions and species nodes within a food web (i.e., who eats whom). This may result from novel species introductions and/or the loss of species. Note that topological rewiring is an extreme case of interaction strength rewiring, where an interaction is added or eliminated from the food web (Bartley et al. 2019; Blanchard 2015)
Node rewiring	A form of asymmetric rewiring that arises from changes in the traits or vital rates of species within a food web (such as reproduction and survival; Cosset et al. 2019), which often occur due to shifts in environmental conditions or resource availability that ultimately affect population demographics
Resilience	The tendency for a system to return to its original state following a perturbation (Donohue et al. 2016; McCann 2000). Here, resilience is characterised as equilibrium stability (the maximum real eigenvalue of the Jacobian matrix, indicating the rate and direction of return to equilibrium) and as non‐equilibrium stability (the coefficient of variation in a population's biomass in the presence of stochastic perturbations)

Asymmetry is a ubiquitous property of nature from molecular to planetary scales (Neumüller and Knoblich 2009; Rathore et al. 2020). Widely recognised examples of asymmetries include the right‐hand twist of the double helix of DNA (Watson and Crick 1953), the migrating eye of flatfish (Campinho et al. 2018), the enlarged claw of a male fiddler crab (Morgan 1923), left or right handedness in humans (Amunts et al. 1996), and differences in air temperature between the northern and southern hemispheres (Feulner et al. 2013). Asymmetry has also been recognised as an important component shaping the structure and dynamics of food webs (de Ruiter et al. 1995; Rooney et al. 2006)—the web of life that feeds biodiversity (Embke and Vander Zanden 2022). In complex food webs, the flow of energy between spatially adjacent habitats, such as benthic and pelagic zones in aquatic systems or detrital and plant‐based carbon channels in terrestrial systems, is often organised in an asymmetric manner that is key to the stability and persistence of the entire ecosystem (Allen‐Perkins et al. 2023; McCann and Rooney 2009; Rooney et al. 2006; Schindler and Scheuerell 2002; Vander Zanden and Vadeboncoeur 2020).

An emerging argument for how anthropogenic pressures asymmetrically rewire food webs involves the reconfiguring of the spatial and temporal structure of ecosystems (Krause et al. 2003; Stouffer and Bascompte 2011). Food webs are structured by distinct habitats with unique abiotic conditions that support different basal resources (e.g., primary producers and detritus), their consumers and the predators that feed on those consumers. Many upper‐trophic level species are highly mobile (i.e., generalist consumers; Table 1; Stiling et al. 2023), traversing habitat boundaries to source energy and resources across a diverse landscape (McCann et al. 2005; Rooney et al. 2008). This mobility allows generalist consumers to rapidly respond to changes in environmental conditions through their behaviour, such that they can avoid habitats with less available resources or that impose negative physiological consequences (McCann and Rooney 2009). However, the exposure of generalist consumers to anthropogenic pressures as they integrate energy and resources from distinct habitat types is also a mechanism for asymmetric rewiring. Anthropogenic pressures may result in such extreme and differential impacts on habitats that some may become inhospitable due to novel physical and biological properties (Tunney et al. 2014). This can result in altered energy flows throughout food webs and restrict options for consumers in a variable world, thereby affecting the *resilience* of whole ecosystems.

An understanding of how human‐driven global change rewires food webs from a spatial perspective is, however, lacking. Such a perspective is required to provide timely scientific advice to those charged with managing nature in the face of global change. To address this gap, we adopt a synthetic approach to present an emerging perspective for asymmetric rewiring of food webs under global change. First, we elucidate the conceptual arguments for asymmetric rewiring by revisiting and building on previous work positing that anthropogenic pressures are broadly impacting habitats and ecosystems differentially, creating novel heterogeneity across spatial scales. We argue that this heterogeneity rewires the spatial organisation of food webs, introducing asymmetries in the *energy pathways* linked by generalist consumers through their *habitat coupling*. We then review the habitat coupling literature to explore the universality of asymmetric rewiring and assay change in the contribution of resources and energy from different habitats through generalist consumers across an array of terrestrial and aquatic ecosystems. To further demonstrate how this process unfolds, we provide a detailed discussion of several cases of asymmetric rewiring from our review. We then conduct simulations using food web models that emulate patterns that emerged from the literature review to assess the potential impacts of asymmetric rewiring on food web structure, ecosystem functions and resilience. The simulations reveal that the rewiring of the spatial structure of food webs has profound consequences for the resilience of ecosystems and key functions, such as species biomass production. We conclude by highlighting future directions to leverage this understanding for management strategies aimed at enhancing and protecting ecosystem resilience and functions in an era of rapid global change.

## 2 The Recipe for Asymmetric Rewiring

**FIGURE 1:**
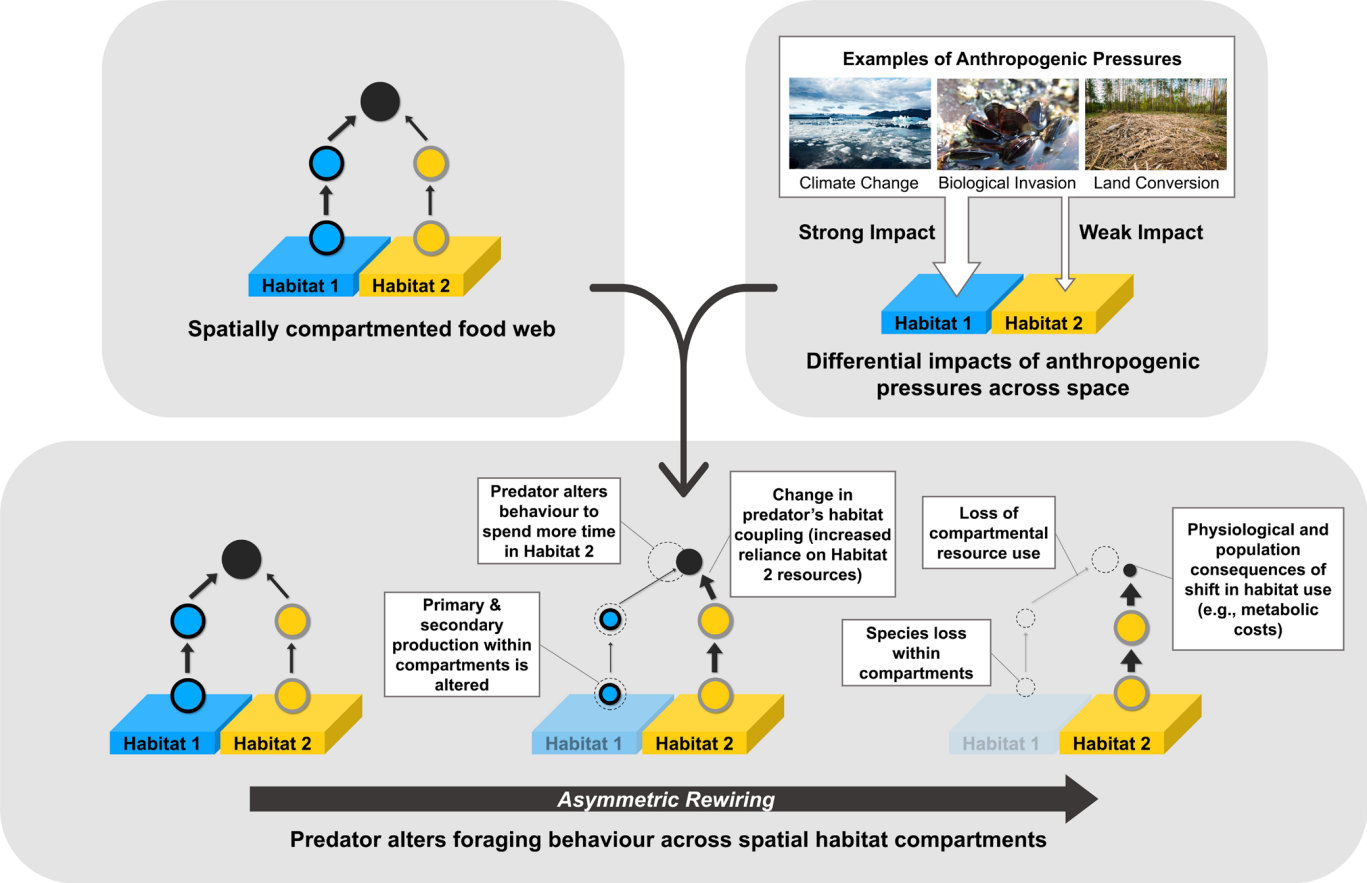
A conceptual diagram of asymmetric rewiring. The spatial compartmentation of food webs combined with the differential impacts of anthropogenic pressures on distinct habitats drives asymmetric rewiring. In spatially compartmented food webs, lower trophic level organisms (blue and yellow circles) occupy distinct habitats (Habitat 1 and Habitat 2) while generalist consumers at higher trophic levels (black circles) forage across habitat boundaries, linking otherwise discrete energy pathways within food webs. As anthropogenic pressures, such as climate change, biological invasions, and land conversion, affect these habitats unevenly, they induce differential impacts that alter the topology (presence of circles and black arrows), interaction strengths (width of black arrows), and the size and quality of the nodes (size of circles) within the food web. For simplicity, the figure shows a generalist top predator coupling resources from two distinct habitats. As pressures intensify, the predator adjusts its foraging, causing shifts in the spatial structure of the food web, resulting in asymmetric rewiring.

Asymmetric rewiring depends on two key ingredients. First, asymmetric rewiring is driven by the inherent spatial compartmentation of food webs (Krause et al. 2003; Moore and Hunt 1988; Yodzis 1982). Lower trophic level organisms tend to occupy discrete habitats (micro‐ and macrohabitats) whereas upper‐trophic level organisms, such as generalist consumers, forage across habitat boundaries (Figure 1; Maitland et al. 2024; Keppeler et al. 2021; Rooney et al. 2008). This creates a nested structure of habitat coupling across space and trophic levels, where generalist consumers acquire and assimilate resources from multiple energy pathways (Vander Zanden and Vadeboncoeur 2002). Notably, while habitat coupling can occur at various trophic levels (Stiling et al. 2023), generalist consumers at higher trophic levels play a crucial role by integrating large areas and adapting their resource use in response to environmental changes and prey availability (Kortsch et al. 2019; Law and Dickman 1998; Tunney et al. 2014). Thus, the ability of generalist consumers to withstand a range of environmental conditions, coupled with differences in the productivity of different habitats, determines the availability and consumption of different resources (Marklund et al. 2018; Ward et al. 1998). This consumption‐based structure sets up the blend of strong and weak energy pathways between consumers and resources from different habitats and is considered an important stabilising attribute of food webs (Rooney et al. 2006).

Second, impacts from anthropogenic pressures are unlikely to exhibit a perfectly symmetrical spatial signature across all scales, leading to differential impacts across spatially distinct habitats or ecosystems (Figure 1). Even spatially general phenomena, such as climate warming, yield varied impacts as they interact with different habitat matrices, which filter anthropogenic pressures differentially (Rathore et al. 2020; Xu and Ramanathan 2012). For example, warming surface waters in a thermally stratified lake affects shallow waters more strongly than cooler deep waters (Pilla et al. 2020). Similarly, invasive species tend to have more or less of an impact on different habitats based on their habitat preferences (Hecky and Hesslein 1995).

Given this, generalist consumers readily respond to changes in environmental conditions or resource availability among the habitats that they couple (Figure 1). Differential impacts of anthropogenic pressures on distinct habitats alter consumers' access to more heavily impacted habitats and/or change the density of resources within these habitats, altering their foraging behaviours and positions within the broader food web. The consequent rewiring occurs asymmetrically and potentially involves changes to food web topology (*topological rewiring*; Table 1), interaction strengths (*interaction strength rewiring*; Table 1), or the traits or vital rates of the species that form nodes within the food web (e.g., metabolic rate and reproduction; Cosset et al. 2019; Srinivasan et al. 2015; *node rewiring*; Table 1). Although we outline three distinct types of rewiring here, it is unlikely that food webs experience topological, interaction strength and node changes independently. Instead, we expect that anthropogenic pressures rewire food webs across multiple dimensions due to their dynamic nature. For example, changes in the traits or characteristics of the food web nodes (e.g., resource population's energy density or consumer's metabolic rate) may cascade into changes in the strength of interactions among the nodes of a food web. Similarly, topological rewiring via the addition or removal of nodes will likely have cascading impacts on the traits within and interaction strengths between remaining nodes.

Under this definition, asymmetric rewiring can manifest in various ways. However, a key manifestation is the alteration of the spatial structure of food webs as generalist consumers that feed among different habitats adjust their degree of habitat coupling (Schindler and Scheuerell 2002). This leads us to argue that differential habitat impacts due to drivers of global change reorganise food webs by altering distinct energy pathways from basal resources to upper‐trophic levels, and are reflected in the behaviour (e.g., habitat use for foraging) of generalist consumer species. As such, empirical estimates of altered habitat coupling among generalist consumers act as assays to document whole food web changes and, thus, the extent of asymmetric rewiring.

## 3 Empirical Observations of Asymmetric Rewiring

Despite the apparent utility of measuring habitat coupling as a spatial indicator of food web alteration under global change (Bartels et al. 2016; Tunney et al. 2014; Manlick et al. 2024), this literature has not been synthesised. To address this gap, we conducted a structured literature search and vote‐count analysis of peer‐reviewed studies and reviewed this dataset as a literature‐based test of the hypothesis that global change asymmetrically rewires the habitat coupling of generalist consumers.

Our literature search focused on studies that quantify the reliance of generalist consumers on spatially explicit energy pathways through one of three approaches: stable isotope analysis, gut content analysis or energetic modelling. Each approach can elucidate habitat coupling but does so in different ways. Therefore, we describe and compare these approaches below in the interest of clarity.

Stable isotope analysis, particularly using stable carbon isotopes, offers a straightforward and rapid means to measure habitat coupling. Carbon is a consistent and major component of biological tissue, and consumers assimilate dietary carbon into their tissues during somatic growth. The ratio of heavy to light carbon isotopes (^13^C/^12^C) differs among primary producers occupying different habitats and is relatively conserved in the tissue of consumers through trophic transfers (Fry 2006). Therefore, carbon stable isotope signatures can indicate which primary producers and, by extension, which habitats are contributing most to consumer biomass through the provision of carbon‐based energy and matter (Peterson 1999; van Oevelen et al. 2006). For instance, if 80% of an aquatic consumer's tissue carbon is traced to nearshore sources, it indicates that a large proportion of assimilated energy and matter was derived from that habitat. While carbon isotopes are more commonly used to trace the spatial origin of resources supporting consumer biomass assimilation, we also considered studies employing other isotopes such as deuterium (^2^H/^1^H) and sulfur (^34^S/^32^S), which operate under the same core principle. These isotopes reflect variation in habitat‐specific source signatures that are retained in consumer tissues and can similarly be used to trace reliance on spatially distinct energy pathways (Amiraux et al. 2023; Brett et al. 2018; Gutgesell et al. 2025). Habitat coupling is typically determined using mixing models or linear interpolation between end‐members representing distinct habitats (Phillips et al. 2014; Post 2002; Stock et al. 2018; Telsnig et al. 2019). Spatial and temporal variation in baseline resource isotope values, as well as uncertainty in trophic fractionation estimates across species, can influence estimates of resource contributions. We therefore considered the study designs and analytical approaches employed when interpreting patterns of habitat coupling derived from isotopic methods (see Appendix S1).

Gut content analysis, on the other hand, provides a high‐resolution snapshot of consumer diet at the time of sampling, wherein prey items can be categorised by their habitat of origin to infer a consumer's energy intake from distinct habitats (Wallace et al. 1997). Habitat coupling is estimated by calculating the proportion of total gut content weight or the relative frequency of prey originating from different habitats (Colborne et al. 2015). Although gut content analysis captures only recent ingestion events and can be limited by the rapid degradation of prey items (Amundsen and Sánchez‐Hernández 2019), it provides concrete evidence of short‐term resource use and longitudinal sampling of gut contents can reveal temporal shifts in consumer diets (Stone et al. 2020). Nevertheless, the source habitats from which prey items are derived are not directly measured as they are in stable isotope analysis but are instead inferred based on prior knowledge of the habitat use and life histories of the prey species consumed.

Energetic modelling approaches, including frameworks such as linear inverse modelling (Gellner et al. 2023), adapted food web energetics modelling (Barnes et al. 2014; Jochum et al. 2021) and Ecopath with Ecosim (Christensen and Walters 2004), are widely used to estimate energy flows in ecological networks (Moore and de Ruiter 2012). These models integrate information on species or functional group biomass, diet preferences and metabolic demands to solve for the distribution of energy flows that balance energy intake and expenditure across species and trophic levels (Barnes et al. 2018; de Ruiter et al. 1993; Jochum et al. 2021; van Oevelen et al. 2010). By quantifying the magnitude and direction of energy flows from habitat‐specific resource pools to consumers, these models allow researchers to infer habitat coupling by calculating the relative contribution of energy from distinct habitats to total energy intake by generalist consumers (Moore and de Ruiter 1991, 2012). However, energetic models generally assume equilibrium (Gellner et al. 2023; van Oevelen et al. 2010), which may not accurately reflect dynamic or fluctuating ecological conditions. In addition, estimates of energy flow can be highly sensitive to the spatial scale of sampling and the resolution of biomass and diet data included in the model. We therefore interpret energy flow estimates as comparative indicators of habitat coupling rather than exact values, which is consistent with best practice for the use of energetic models in food web studies (Jochum et al. 2021).

To synthesise the habitat coupling literature, we categorised anthropogenic pressures based on whether they increased, decreased or did not change the reliance of higher‐order generalist consumers (e.g., secondary consumers and predators) on resources from multiple habitats. We used the authors' descriptions to assess the spatial distinctness of habitats as well as their reported results to determine whether generalist consumers shifted their reliance on resources from these habitats. We also conducted a cursory review of each study to ensure consistency with our definition of energy pathways and habitat coupling (Table 1). Following an extensive search and screening process, we identified 85 studies that met our criteria (Appendix S1).

**FIGURE 2:**
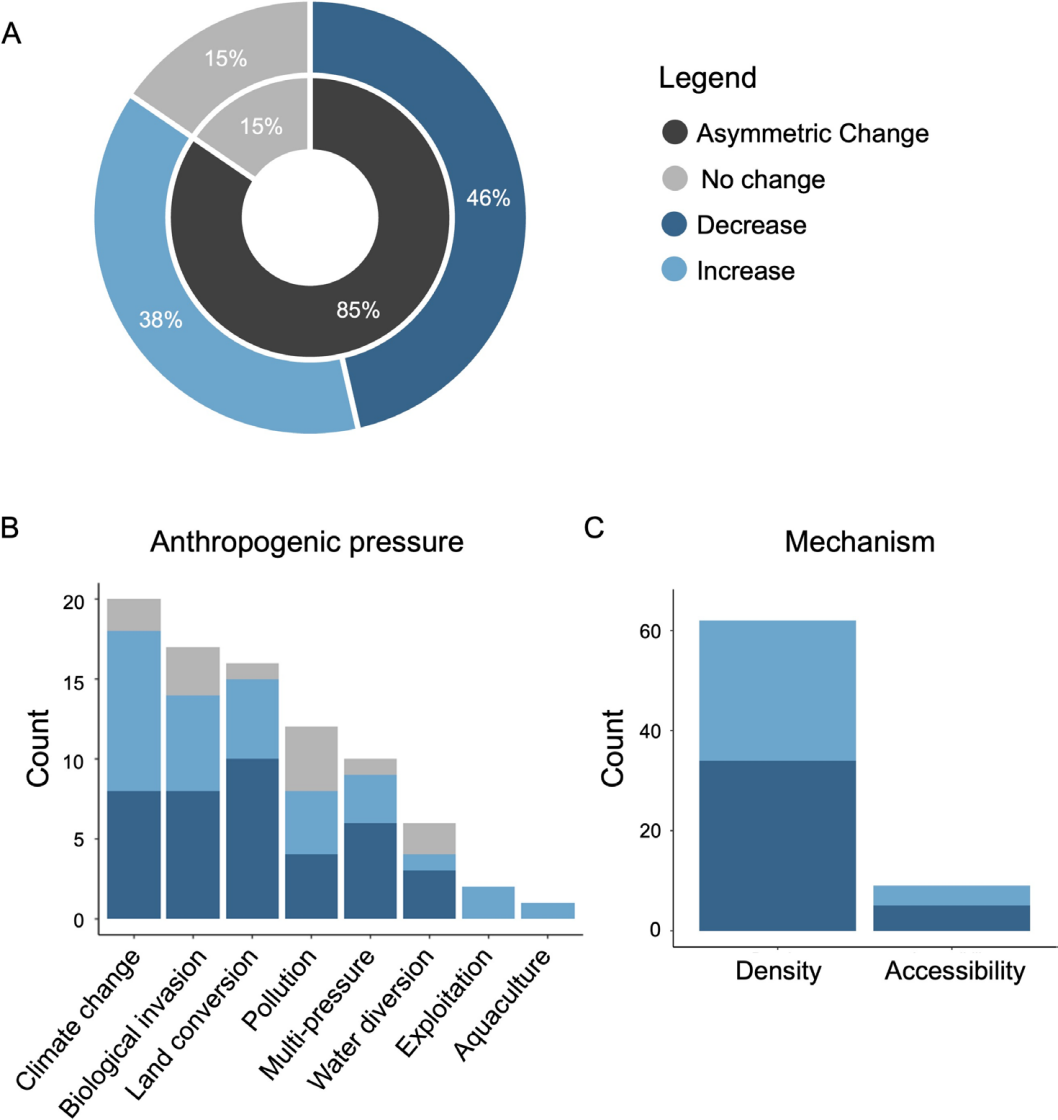
Literature review of studies (total *n* = 85) that examine generalist consumer habitat coupling in response to anthropogenic pressures. (A) A high percentage of studies found asymmetric change in habitat coupling (black) versus no change (grey). Fewer studies report increases in habitat coupling (light blue) compared to decreases (dark blue). (B) A comparison of the number of studies that show an increase (light blue), decrease (dark blue) or no change (grey) in habitat coupling grouped by anthropogenic pressure. (C) A comparison of the number of studies that investigated the direction of change (increase: light blue and decrease: dark blue) in habitat coupling associated with consumer habitat accessibility and habitat resource density.

We found that 72 of 85 studies (85%) detected a response in habitat coupling to the differential impacts of anthropogenic pressures on distinct habitats (Figure 2A [asymmetric change]; Table S1). Approximately 54% of those (39 out of 72 studies, 46% of all studies examined) reported a decrease in habitat coupling, meaning that generalist consumers responded to anthropogenic pressure by increasing the intake of resources from one dominant habitat and decreasing reliance on resources from additional habitats (Figure 2A). An increase in habitat coupling spread across multiple habitats was reported in 39% of studies (33 of 85 studies), while no change in coupling in response to anthropogenic pressure was found in 15% of studies in our review (13 out of 85 studies; Figure 2A).

The direction of habitat coupling responses to anthropogenic pressure appears to depend on a consumer's initial resource use and behavioural flexibility. For instance, consumers that rely heavily on resources from a single habitat under historical or ‘natural’ conditions may diversify their resource use under pressure, thus increasing habitat coupling (McKinney et al. 2013; Wang et al. 2016). Conversely, consumers that acquire resources from multiple habitats more evenly in the absence of anthropogenic pressure may reduce their use of resources derived from more impacted habitats, thus decreasing habitat coupling (England and Rosemond 2004; Miehls et al. 2009). In both cases, anthropogenic pressures differentially impact habitats, and energy pathways are asymmetrically affected, but the direction in which habitat coupling changes differs. Generalist consumers may also maintain a preference for prey from particular habitats, and as a result, habitat coupling may remain unchanged despite differential changes to habitat conditions (e.g., Roon et al. 2016).

We grouped and assessed studies based on the type of anthropogenic pressure investigated to explore whether certain anthropogenic pressures are associated with consistent patterns in the direction of change in habitat coupling. Studies that focused on climate change were most numerous (*n* = 21), followed by biological invasions (*n* = 17) and land conversion (*n* = 16; Figure 2B; Table S1). Climate change most often led to an increase in habitat coupling (*n* = 11; 52% of studies examining this driver of global change), whereas biological invasions showed a tendency to decrease habitat coupling by consumers (*n* = 8; 47% of studies examining this driver of global change). Land conversion tended to result in a decoupling of habitats (*n* = 10; 63% of studies examining this driver of global change).

We investigated two common mechanisms that influence the availability of prey resources to consumers, namely the density of resources and their accessibility. Changes in habitat coupling due to altered resource density occur when the productivity (primary or secondary production) of distinct habitats is affected by anthropogenic pressures (Hunt et al. 2020). Whereas changes in habitat coupling due to accessibility occur when the fraction of resource density within habitats that a consumer can acquire becomes limited. This limitation can arise, for example, when environmental conditions within a habitat become so physiologically stressful that a consumer avoids foraging in that habitat (Tunney et al. 2014). We found that resource density is a more broadly identified mechanistic driver of change in habitat coupling in the literature compared to changes in the accessibility of prey in a particular habitat (86% and 14% of studies that reported a change in habitat coupling, respectively), but that both increases and decreases in consumer habitat coupling were identified in both categories (Figure 2C).

The majority of studies captured in our review employed stable isotope analysis (*n* = 64, 75%), while studies employing gut content analysis (*n* = 9, 11%) and energetic modelling (*n* = 12, 14%) were less common. This pattern likely reflects the timing of habitat coupling's integration into ecological theory, which coincided with the widespread adoption of stable isotope methods as a standard tool for tracing energy flows across spatial boundaries (McCann et al. 2005; Schindler and Scheuerell 2002; Vander Zanden et al. 1999; Vander Zanden and Vadeboncoeur 2002). However, studies applying energetic modelling to examine spatial energy flows appear to have become more frequent in recent years, with 58% of the energetic modelling studies included in our review (7 of 12) published since 2020.

Aquatic ecosystems dominated the habitat coupling literature, accounting for 78% (66 of 85) of the studies analysed. Within aquatic systems, lentic ecosystems (e.g., lakes; *n* = 28) were most numerous, followed by lotic systems (e.g., rivers; *n* = 23) and marine systems (*n* = 15). Habitat coupling appears comparatively underexplored in marine environments relative to freshwater systems despite their representing a substantial portion of global biodiversity and food supply (FAO 2022) and their being heavily studied for other aspects of ecosystem structure and function (Gamfeldt et al. 2015; O'Connor and Crowe 2005). Given the existence of large‐scale government surveys in marine environments (Cook and Bundy 2012), fisheries monitoring programmes (Boenish et al. 2020) and extensive marine ecological datasets (Edwards et al. 2010; Southward et al. 2005), developing research on habitat coupling in these systems presents a tractable and important opportunity to better understand changes to spatial energy pathways.

Terrestrial ecosystems remain similarly underrepresented in the habitat coupling literature, comprising the focus of only 22% (19 of 85) of studies in our review. Stable isotope analysis (the most common approach in our review) can be difficult to apply in this context due to limited fine‐scale variation in baseline isotope values of terrestrial organisms (Kaplan et al. 2002). Advancing methods to resolve this, such as compound‐specific stable isotope analysis (O'Brien et al. 2005), improved models for resolving consumer diets using stable isotopes (Arnoldi et al. 2024), and integrating acoustic or radio telemetry tracking approaches with isotopic data (Caron‐Beaudoin et al. 2013), will be key to elucidating how terrestrial food webs respond to global change. Expanding research across both marine and terrestrial systems is essential to unpack the diversity of consumer responses and mechanisms driving the rewiring of food webs.

**FIGURE 3:**
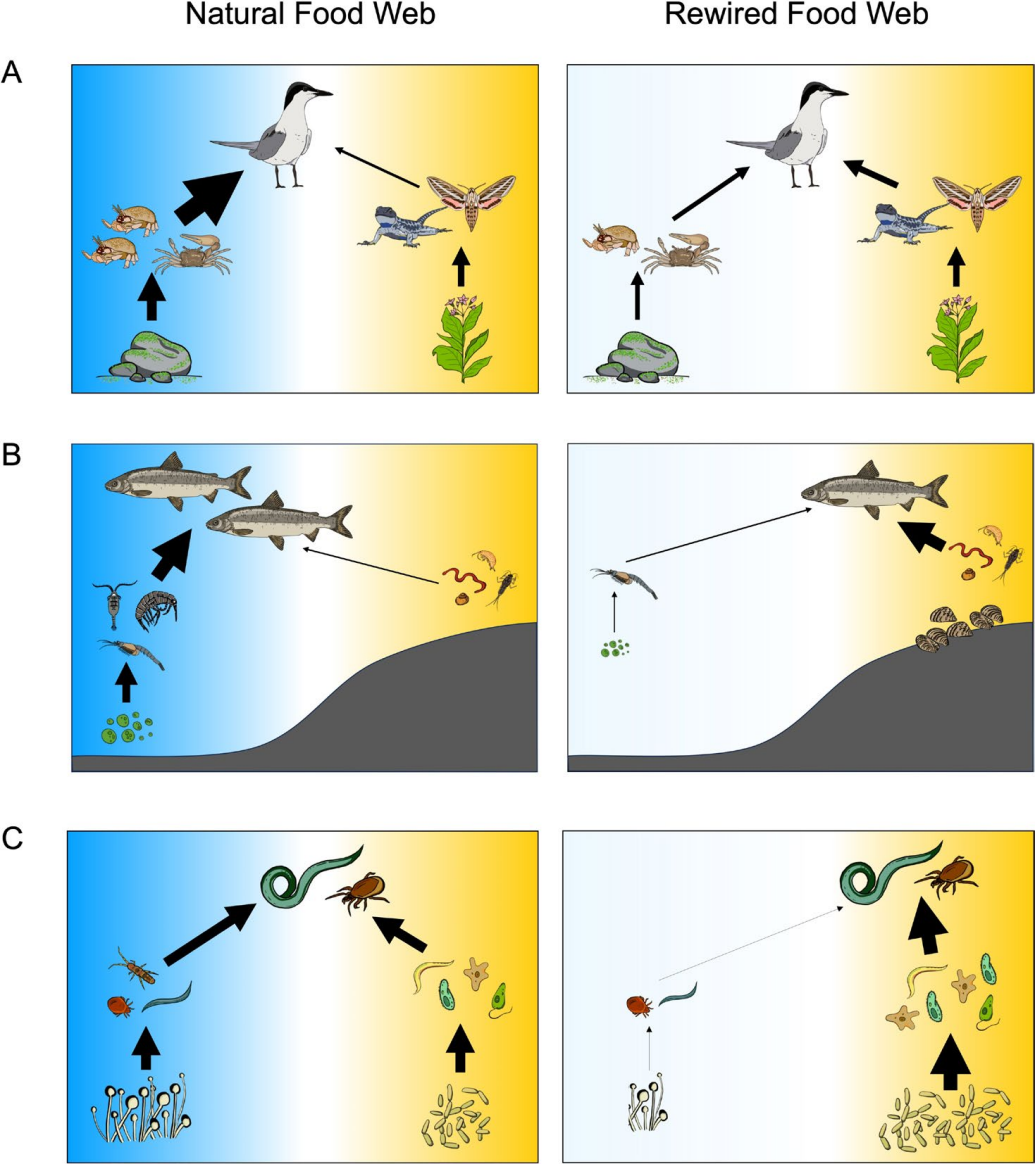
Differential impacts from anthropogenic pressures on habitats (blue and yellow shading indicates different habitats) alter energy pathways from basal resources to top predators. In all examples, the blue habitat represents the more impacted habitat. Arrows represent the strength of consumptive interactions and indicate relative changes in resource contributions to generalist consumers. (A) Food webs during cool (natural) and warm (rewired) years in South San Diego Bay and the Tijuana River Estuary, southern California. Climate‐driven warming events (ENSO and the Pacific ‘blob’) reduce the availability of the Gull‐billed tern's preferred aquatic (blue) prey, *Emerita analoga* , leading terns to shift toward terrestrial (yellow) prey and decreasing the relative contribution of aquatic resources to their diet (Goodenough et al. 2023). (B) Laurentian Great Lakes food webs before (natural) and after (rewired) the invasion of dreissenid mussels. Mussels redirect pelagic (blue) primary production to nearshore benthic (yellow) zones, increasing lake whitefish reliance on less‐preferred nearshore prey and reducing both body condition and population size in Lake Huron (Fera et al. 2017; Rennie et al. 2009a, 2009b). (C) Soil food webs in grasslands (natural) and intensive agricultural (rewired) systems. Agricultural practices such as tillage and fertiliser input increase bacterial (yellow) activity in the soil matrix, decreasing the relative reliance of predatory soil organisms (e.g., predatory nematodes) on the slower‐cycling fungal (blue) pathway (Moore and Mueller 2024). This shift reduces the C:N ratio of detritus and alters net primary productivity of plant communities, affecting soil carbon sequestration. Artist Credit (organism illustrations): Dr. Carling Bieg.

There were three notable spatially coupled food web types that were well represented in our review: aquatic–terrestrial energy pathways, nearshore–offshore energy pathways and soil bacterial–fungal energy pathways. In what follows, we explore several examples of asymmetric rewiring within these broad food web types. In each example, we explain how anthropogenic pressures have differentially impacted distinct habitats (micro‐ and macrohabitats) and how generalist consumers adjust their foraging behaviour and resource use in response. We also highlight the three most common anthropogenic pressures identified in our review (climate change, biological invasion and land conversion) with specific cases shown in Figure 3A–C.

### 3.1 Asymmetric Rewiring of Aquatic–Terrestrial Energy Pathways

Food webs at the interface of aquatic and terrestrial systems are supported by reciprocal resource exchanges between these distinct habitats (Schindler and Smits 2017; Soininen et al. 2015). Terrestrial habitats contribute allochthonous carbon to aquatic habitats through leaf litter, woody debris and terrestrial invertebrates, providing the foundation for aquatic decomposers and detritivorous macroinvertebrates (Wallace et al. 1997). Aquatic habitats, in turn, support the emergence of aquatic macroinvertebrates, which provide prey subsidies to riparian predators (Baxter et al. 2005; Burdon and Harding 2008; Paetzold et al. 2006; Sabo and Power 2002). For example, riparian lizards derive a substantial portion of their annual energy budget from aquatic macroinvertebrates during peak emergence (Sabo and Power 2002). This timing aligns with periods when terrestrial prey availability is low, and later in the growing season, terrestrial invertebrates and leaf litter inputs increasingly subsidise aquatic food webs (Kawaguchi et al. 2003; Kawaguchi and Nakano 2001). Such seasonal complementarity in energy flows across habitats can stabilise consumer populations by reducing fluctuations in resource availability throughout the year (Huxel et al. 2002; Polis et al. 1997; Scott and Wesner 2010; Takimoto et al. 2002).

Anthropogenic pressures that differentially impact aquatic and terrestrial habitats are disrupting the timing and strength of these seasonal fluxes (Larsen et al. 2016). For example, deforestation and riparian canopy loss reduce terrestrial detrital inputs and invertebrate in‐fall to stream environments, leading generalist fish species such as creek chub ( *Semotilus atromaculatus* ) to shift reliance away from terrestrially derived prey (Champagne et al. 2022). Simultaneously, eutrophication from agricultural runoff may increase instream primary production (Dodds and Smith 2016; Dunck et al. 2015; Tromboni et al. 2019), further intensifying consumer reliance on aquatic energy pathways and leading to top‐down control of aquatic resources (Gutgesell et al. 2025).

Pollution and water diversion are also important drivers of altered aquatic–terrestrial coupling by consumers. In riparian habitats impacted by livestock grazing and manure inputs to adjacent streams, orb‐weaver spiders (*Araneidae*) decreased their reliance on aquatic resources and shifted toward greater use of terrestrial prey, reflecting a reduction in aquatic–terrestrial coupling driven by declines in aquatic resource availability in polluted streams (Hunt et al. 2020). In contrast, in urbanised environments, riparian orb‐weaving spiders incorporated up to 30% more aquatic prey into their diets compared to spiders in less urbanised sites, a shift attributed to both reduced terrestrial prey availability and the increased abundance of pollution‐tolerant emergent aquatic macroinvertebrates, such as chironomids (Kelly et al. 2019). Similarly, water diversion through activities such as damming can suppress terrestrial detrital inputs while promoting algal growth, leading aquatic consumers to shift their reliance toward resources from autochthonous (aquatic) energy pathways (de Guzman et al. 2024; Roussel et al. 2023).

Climate change can similarly impact the aquatic–terrestrial coupling by consumers. A natural warming experiment simulating projected climate impacts on stream food webs found aquatic macroinvertebrates assemblages increasingly relied on autochthonous (aquatic) production as stream temperatures rose. The authors argue this is likely due to the energetic advantages of consuming energy‐dense aquatic resources in warmer environments where metabolic costs are higher (Jackson et al. 2024). In coastal marine ecosystems, Gull‐billed Terns ( *Gelochelidon nilotica* ) in southern California historically relied heavily on the intertidal crustacean *Emerita analoga* , which comprised up to 76% of their diet before 2013 (Goodenough et al. 2023). However, from 2014 to 2016, a combination of marine heatwave events (the warmwater pacific ‘blob’ and ENSO) caused *Emerita* densities to decline by more than 90% in some areas, leading to a dramatic shift in marine prey availability (Figure 3A). In response, terns shifted their diet toward terrestrially derived prey (e.g., lizards, flying insects, birds) and more thermally tolerant aquatic crustaceans, such as *Leptuca crenulata* and *Pleuroncodes planipes*, resulting in an increase in aquatic–terrestrial habitat coupling during warmer‐than‐average years (Figure 3A; Goodenough et al. 2023).

Changes in resource densities among aquatic and terrestrial energy pathways not only modify interaction strengths among food web members, but also influence their traits, for instance by affecting the growth rates, body sizes and metabolism of fish (Atlas et al. 2013; Eros et al. 2012). Such responses reflect node rewiring, where anthropogenic pressures appear to be altering consumer physiology and resource quality (Larrañaga et al. 2023; Merckx et al. 2018). Consequently, the differential impacts of anthropogenic pressures on terrestrial and aquatic habitats lead to behavioural and demographic shifts in organisms, rewiring energy pathways within these interconnected food webs.

### 3.2 Asymmetric Rewiring of Nearshore–Offshore Energy Pathways

The nearshore and offshore habitats of freshwater lakes and marine environments differ in light, temperature, nutrient dynamics and community composition, collectively supporting diverse assemblages of primary producers and consumers (Corcoran and Shipe 2011; Gorman et al. 2012; Wang et al. 2019). Generalist consumers, particularly mid‐ and upper‐trophic level fish, integrate energy from both nearshore prey, such as benthic macroinvertebrates and offshore prey, like zooplankton (Vander Zanden et al. 2005; Vander Zanden and Vadeboncoeur 2002). Anthropogenic pressures that impact aquatic ecosystems often affect nearshore and offshore habitats differently, yielding significant rewiring of nearshore–offshore energy pathways.

For example, the invasion of zebra mussels ( *Dreissena polymorpha* ) in the Laurentian Great Lakes topologically rewired food webs by establishing novel interactions among mussels, phytoplankton and benthic biofilms. By filtering phytoplankton and sequestering nutrients in nearshore benthic habitats, zebra mussels have redirected energy away from offshore primary production (Hecky et al. 2011). In response, cold‐water consumers such as lake whitefish ( *Coregonus clupeaformis* ) have shifted their foraging from zooplankton to the less energy‐dense mussels, which coincided with declines in body condition and somatic growth (Figure 3B; Fera et al. 2017; Rennie et al. 2009a, 2009b). In this case, topological, interaction strength and node rewiring have collectively altered the spatial structure of the Laurentian Great Lakes food webs, as shifts in food web members and the availability of compartmental resources have reshaped consumer traits and energy intake (Rennie et al. 2009a, 2009b).

The coupling of nearshore–offshore energy pathways by aquatic consumers has been particularly well studied in the context of climate change. In North temperate freshwater lakes, nearshore habitats are warming more rapidly than offshore habitats, creating distinct thermal zones that influence predator habitat use and foraging behaviour (Cline et al. 2013). Lake trout ( *Salvelinus namaycush* ), a cold‐adapted top predator, have been observed to reduce their use of nearshore habitats as surface waters warm, instead assimilating more energy from offshore resources, such as zooplankton and pelagic forage fish (Guzzo et al. 2017; Tunney et al. 2014). For instance, Guzzo et al. (2017) found that longer summers increased nearshore–offshore coupling, as lake trout relied almost entirely on resources from nearshore habitats in years with shorter summers (~90% of resource assimilated from nearshore pathway) but shifted their resource use to rely on offshore resources in years with longer, more thermally restrictive summers (~50% of resources assimilated from nearshore pathway). Similarly, Tunney et al. (2014) found that across a spatial gradient of North temperate lakes, lake trout reduced their reliance on nearshore prey due to reduced access to thermally stressful nearshore habitats in warmer lakes.

In marine ecosystems, top predators such as polar bears ( *Ursus maritimus* ) are shifting their foraging patterns in response to climate‐driven sea‐ice loss (Johnson et al. 2019). Over the past three decades, changes in seasonal sea‐ice dynamics in Eastern Greenland have reduced polar bear access to ice‐associated nearshore prey species (e.g., ringed seals; *Pusa hispida* ) and led to greater reliance on pelagic sub‐Arctic species (e.g., hooded seals; *Cystophora cristata* ; McKinney et al. 2013), thereby strengthening their nearshore–offshore habitat coupling. Importantly, sub‐Arctic prey tend to carry higher loads of persistent organic pollutants and mercury compared to Arctic species (Letcher et al. 2010; Sonne 2010), and the growing reliance on offshore prey has contributed to increased contaminant levels in polar bear tissues over time (McKinney et al. 2013). As contaminant burdens accumulate, there is increasing concern over negative impacts on polar bear immune and reproductive health, potentially altering key traits such as reproductive rates and rewiring the nodes and interactions within Arctic food webs (Stirling and Derocher 2012). Similarly, warming in Cumberland Sound has facilitated the northward range expansion of capelin ( *Mallotus villosus* ), a pelagic forage fish, into Arctic waters, supporting convergent foraging by multiple predator species, including beluga whales ( *Delphinapterus leucas* ), Arctic charr ( *Salvelinus alpinus* ) and Greenland halibut ( *Reinhardtius hippoglossoides* ) (Ulrich and Tallman 2021; Yurkowski et al. 2018). The topological rewiring of the food web has altered prey availability in the offshore habitat, with reductions in trophic niche breadth and functional diversity among predators.

### 3.3 Asymmetric Rewiring of Soil Bacterial–Fungal Energy Pathways

Soil food webs are among the most diverse and complex food webs on Earth (Wagg et al. 2014). To simplify their study, soil organisms are often categorised into functional groups (*sensu* Hunt et al. 1987) based on size, spatial habitat use and ecological roles, including microflora (bacteria and fungi), microfauna (e.g., nematodes and protozoans), mesofauna (e.g., mites and springtails) and macrofauna (e.g., earthworms and termites; Pritchard 2011). Microflora form the base of these webs by decomposing plant‐derived organic matter and facilitating the flow of carbon to higher trophic levels. Two primary energy pathways structure the flow of this detrital carbon: the bacterial pathway, which processes nutrients and carbon on rapid timescales, and the fungal pathway, which supports slower, more carbon‐retentive trophic chains (Moore and de Ruiter 1991). These pathways differ in energy efficiency and nutrient dynamics, with many soil mesofauna and macrofauna consuming both fungal and bacterial resources, thus coupling distinct microbial energy pathways (Moore et al. 2005).

Land‐use change and intensive agriculture are known to alter the structure of soil food webs by disrupting fungal hyphal networks (Hendrix et al. 1986; Holland and Coleman 1987). These disruptions can shift the dominance of bacterial and fungal energy availability, causing generalist consumers to rely on bacterial pathways to a greater extent than on fungal pathways on farms with conventional agricultural practices, such as tillage and fertiliser treatments (Figure 3C; Moore 1994; Moore and Mueller 2024). In a recent study, Potapov et al. (2024) found that the conversion of tropical rainforest to monoculture plantations led to an almost twofold increase in the ratio of bacteria‐to‐fungi energy transferred to soil arthropods in oil palm monocultures. This shift was marked by a greater reliance on bacterial pathways compared to intact rainforest plots, resulting in increased coupling of bacterial and fungal energy pathways by soil arthropods in plantations (Potapov et al. 2024). Notably, a large‐scale study examining changes in soil food web structure across a broad geographical region in Europe found that agricultural intensification reduced the biomass of most feeding groups in the soil food web, without affecting the relative biomass of bacterial and fungal resource pools, suggesting a symmetrically negative impact on these distinct energy pathways (de Vries et al. 2013).

Climate change has been observed to influence the dominance of bacterial and fungal energy pathways in soil food webs. Mesocosm experiments have shown that warming can induce shifts in the spatial distribution of soil organisms, such as the disappearance of surface‐dwelling earthworms (epigeic worms) and a deeper distribution of enchytraeids (Briones et al. 2009). These shifts were accompanied by an increase in fungivorous mites, suggesting a transition from a bacterial‐ to a fungal‐driven food web with warming (Briones et al. 2009). Long‐term climate changes and experimental warming led to similar changes among food webs in Arctic tundra and boreal ecosystems (Manlick et al. 2024). Here, predatory shrews exhibited significant increases in fungal carbon assimilation over a period of warming, while bacterial carbon contributions remained consistently low across species (Manlick et al. 2024). These changes have broad implications for carbon processing within soils, as microbial pathways mediate energy flow across trophic levels and play a critical role in carbon storage and nutrient cycling in terrestrial ecosystems (Moore et al. 2005).

## 4 Ecosystem Consequences of Asymmetric Rewiring

We have shown that anthropogenic pressures associated with global change are broadly restructuring food webs through the process of asymmetric rewiring. This is concerning because central to ecological theory is the understanding that the structure of ecosystems profoundly influences their functions and dynamic properties, such as resilience (Pauly et al. 1998; Petchey et al. 1999; Sackett et al. 2010). Thus, global change is not only rewiring (restructuring) food webs but also, critically, re‐functioning ecosystems and impacting their ability to recover from natural and human‐driven perturbations.

**FIGURE 4:**
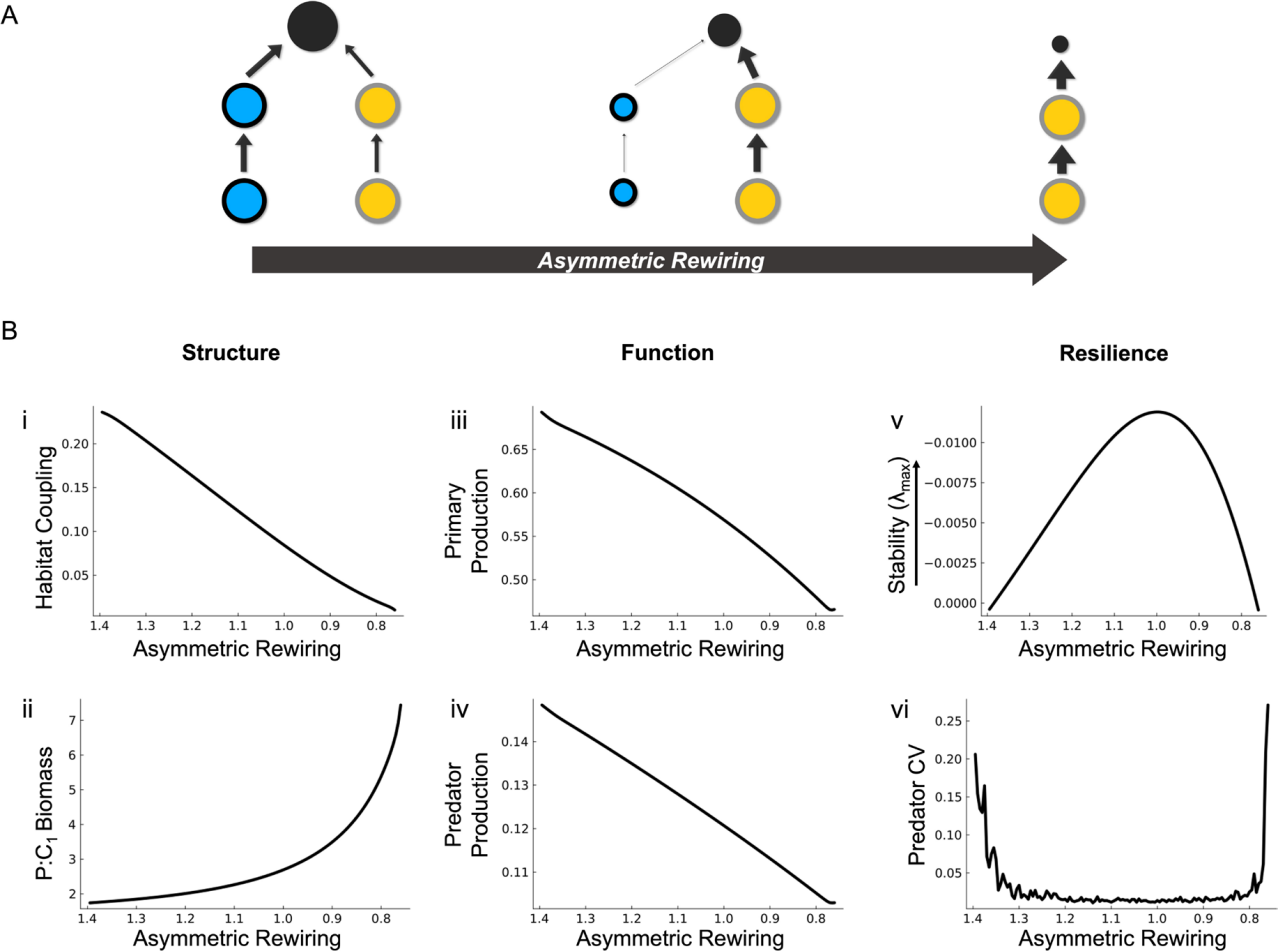
Asymmetric rewiring effects on food web structure, ecosystem functions and resilience depicted in (A) a summary conceptual diagram and (B) outputs of a simulation experiment. (A) A top predator (black circle, size of circle depicts trophospecies biomass) couples prey from Habitat 1 (blue circles) and Habitat 2 (yellow circles) but has a slight preference for prey in Habitat 1 (arrows reflect the presence and strength of consumptive interactions). As anthropogenic pressure increases (moving from left to right), the structure of the food web is rewired, shifting from a five species coupled food web to an uncoupled three species food chain. (B) Outputs of a simulation experiment show that (i) as asymmetric rewiring increases along the *x*‐axis, the predator's degree of coupling is reduced, (ii) the predator to consumer biomass ratio (P:C1) increases exponentially in Habitat 1. Concomitant with these changes in structure, functions such as primary production (iii) and top predator production (iv) decrease, while (v) the equilibrium stability (maximum real eigenvalues) and predator's CV in a stochastically perturbed system (vi) are unimodal.

To explore the mechanisms underlying this structure–function–resilience relationship in the context of global change, we first refer to the processes driving asymmetric rewiring. The compartmentalised energy pathways depicted in Figure 1 form the structural response framework to changing habitat conditions. Importantly, the consequences of food web structure for the stability of food webs have been well studied in the theoretical literature (Moore and Hunt 1988; Stouffer and Bascompte 2011; Teng and McCann 2004; Yodzis 1982). Building on this foundation, we use a simple model to recreate a gradient of food web responses to differential impacts of anthropogenic pressures on habitats, providing a conceptual entry point to how asymmetric rewiring simultaneously alters the structure, functions and dynamical properties (i.e., resilience) of ecosystems (Figure 4).

Our model food web includes two basal resource *trophospecies* (R_1_ and R_2_) with logistic population growth that occupy distinct habitats, and two consumer trophospecies, C_1_ and C_2_, that forage exclusively on R_1_ and R_2_, respectively. The top predator trophospecies (P) forages on both C_1_ and C_2_, thereby coupling Habitats 1 and 2, but does not consume basal resources directly. The predator's preference for the consumer trophospecies (C_1_ and C_2_) is modelled using a density‐dependent behavioural response, with a slight initial preference for C_1_ over C_2_, reflecting natural asymmetries in food web structure (Rooney et al. 2006). With this model, we conduct an experiment where we incrementally impose a differential change in the productivity of Habitat 1 relative to Habitat 2. Specifically, we reduce R_1_'s carrying capacity (*K*_1_) while holding R_2_'s carrying capacity (*K*_2_) constant (see detailed description of experimental approach in Appendix S1).

The result from our experiment is a combination of structural changes in the food web. Habitat coupling is reduced (Figure 4B, i), while the predator to consumer biomass ratio in Habitat 1 (P:C_1_) becomes inflated (Figure 4B, ii) due to the dwindling resource supply in Habitat 1 (i.e., C_1_ declines because of reduction in R_1_ availability). The changing functions follow from these structural changes, such that declining resource carrying capacity in Habitat 1 across the asymmetric rewiring gradient reduces total primary production (Figure 4B, iii). With the energy pathway in Habitat 1 lost due to the decline in C_1_, energy available to the predator becomes limited (Figure S3a) and its secondary production decreases (Figure 4B, iv).

Resilience, measured both as equilibrium (maximum real eigenvalues; Figure 4B, v) and non‐equilibrium (predator coefficient of variation (CV); Figure 4B, vi) dynamical stability, follows the well‐known hump‐shaped relationship across the gradient of asymmetric rewiring (see Appendix S1 for parameters; Rooney et al. 2006). Specifically, the greatest dynamical stability occurs at intermediate levels of habitat coupling. Instability arises at both extremes, either when energy pathways leading to the top predator are both strong (i.e., strong habitat coupling) or when one pathway dominates entirely (i.e., a single food chain; Rooney and McCann 2012). At intermediate coupling, the predator can shift its foraging between habitats in response to changes in prey density, which allows the two prey populations (C_1_ and C_2_) to fluctuate out of phase (McCann et al. 2005). This creates a more stable energy supply for the predator over time and helps to dampen population fluctuations. In contrast, when both pathways are strong, the predator tracks both prey synchronously, removing the stabilising effect of habitat structure. Similarly, when the predator relies on a single energy pathway, it lacks an alternative energy source and may deplete resources in that habitat. In both cases, resilience is reduced compared to systems with intermediate habitat coupling, where the structure allows for some flexibility in energy use and more stable predator dynamics.

Our experiment serves as a useful tool for understanding how asymmetric rewiring could unfold, but it is important to recognise that emergent changes in ecosystem function and resilience in response to anthropogenic pressures are expected to depend on the mechanisms driving novel asymmetries among food webs. In our example, the total energy available to the top predator is reduced. In such cases (e.g., emerald shiners [ *Notropis atherinoides* ] and rainbow smelt [ *Osmerus mordax* ] losing access to benthic resources during periods of hypoxia; Stone et al. 2020), ecosystems will likely experience instabilities and loss of function caused by populations being reduced to dangerously low mean densities (Gellner et al. 2016; Ward et al. 2023). However, differential habitat impacts may similarly affect food web structure while the total energy available to the top predator increases. For example, anthropogenic pressures that deplete resources in Habitat 1 while dramatically increasing resource availability in Habitat 2 (e.g., aquatic‐based resource availability for consumers in streams experiencing increased nutrient runoff after riparian removal; Champagne et al. 2022) would lead to a single, dominant food chain with strong interactions among C_2_ and P that exhibit oscillatory dynamics (Gutgesell et al. 2025; Hastings 1988; McCann 2000). Though the accessibility of newly abundant resources in Habitat 2 may be constrained by behavioural or physiological limitations of generalist consumers. For instance, cold‐water species such as Lake Whitefish ( *Coregonus clupeaformis* ) may be unable to exploit nearshore or benthic production due to thermal constraints, even if the density of those resources increases (McNickle et al. 2006).

The theoretical literature is replete with spatially explicit food web models (DeAngelis et al. 1989; Huxel et al. 2002; Post et al. 2000; van Oevelen et al. 2010), and our experiment applies this framework to conceptually explore how anthropogenic pressures that differentially impact habitats drive the rewiring process outlined in Figure 1. Notably, we find that changes in the structural symmetry of food webs influence ecosystem functions and resilience in predictable, but not necessarily unidirectional, ways. While systems may initially respond to certain habitat changes by becoming more symmetrical (i.e., moving toward a more strongly coupled food web), we argue that this is a transient state. If sustained anthropogenic pressure disproportionately affects one habitat, the system will eventually shift toward strong asymmetry, characterised by the loss of energy pathways among the most impacted habitats and reduced resilience. A primary mechanism underlying this reduction in resilience is the erosion of habitat options, which limits the ability of generalist consumers to respond to variation in resource availability (McMeans et al. 2016; Schindler et al. 2013). Further, and importantly, these findings underscore the utility of key food web structures (e.g., habitat coupling) as indicators for monitoring ecosystem functions and resilience in the face of global change, reinforcing the necessity to integrate these insights into broader ecological management and conservation strategies.

## 5 Conclusions

Our synthesis reveals that food webs are becoming asymmetrically rewired across ecosystems globally. This occurs as generalist consumers alter their reliance on spatially distinct resources in response to the differential impacts of anthropogenic pressures on habitats. In cases where the impacts of anthropogenic pressures are strong and disproportionate, some habitats may become inhospitable or depleted of resources. Consequently, generalist consumers that acquire resources from multiple habitats may ultimately lose access to entire energy pathways, leading to adverse effects on their secondary production and, per theory, the resilience of whole food webs (McCann et al. 2005). Such a loss removes key ‘switch points’ that mobile generalist consumer species use to flexibly adapt their behaviour in response to perturbations (McMeans et al. 2016). We note that these global change and food web asymmetries likely appear across scales (e.g., soil microhabitats; Moore 1994, to entire hemispheres; Imrit and Sharma 2021), suggesting that eroding these stabilising processes concurrently may yield deleterious effects for organisms across trophic levels, affecting ecosystems in profound and potentially irreversible ways.

Based on our literature review, diverse responses in consumer habitat coupling occur across anthropogenic pressures and ecosystems globally. Climate change tended to increase consumer habitat coupling, which may arise due to decreased access to a preferred prey (Guzzo et al. 2017) or increased production in a previously unproductive habitat (Wang et al. 2016). Invasion tended to decrease habitat coupling and may reflect instances when prolific invasive species shift production to a single energy pathway (Gobel et al. 2023; Wood et al. 2017). The conversion of naturally complex habitats to more simplified ecosystems is consistent with land conversion tending to decrease habitat coupling in our review (Stenroth et al. 2015). Importantly, we find that asymmetric rewiring may temporarily increase food web symmetry by increasing the degree of consumer habitat coupling toward an intermediate proportion where habitats are equally coupled in response to anthropogenic pressures (as it has in 39% of the studies examined here). However, because anthropogenic pressure is often sustained and directional, increased symmetry is a transitional state as the dominance shifts from one habitat to another. Eventually, the asymmetric impacts of anthropogenic pressures are likely to result in the loss or near‐loss of entire energy pathways in the most affected habitats.

Our synthesis of asymmetric rewiring provides a conceptual framework to link human activities and associated changes in the properties of ecosystems. While the empirical evidence presented here is compelling, our review revealed substantial gaps in knowledge for specific anthropogenic pressures, including pollution, eutrophication and hydrological alteration. These understudied areas limit our ability to fully generalise the patterns of asymmetric rewiring across all types of anthropogenic impact. Understanding and identifying the mechanisms driving asymmetric rewiring is also vital for leveraging this perspective in policy and management operations during an era of rapid global change. Integrated monitoring programmes that follow the fate of differential habitat impacts can aid in identifying changes in habitat conditions (e.g., using sensors to detect abiotic or biotic conditions) and energy flow throughout food webs (e.g., using stable isotope analysis), and thus delineate mechanisms of asymmetric rewiring.

Collectively, such monitoring systems promise the potential for a suite of early warning signals of looming change, including alterations to the spatial structure of food webs, which can be employed in the mitigation and restoration of global ecosystems. To this end, resource managers are developing strategies to adapt to global change that use frameworks and tools that are amenable to integrating the concept of asymmetric rewiring (Antwi et al. 2024; Boyce et al. 2021; Schindler and Hilborn 2015). For example, considering the spatial scale and intensity of human pressures on habitats may prove critical for ecosystem approaches to managing harvest or prioritising habitats for protection or restoration. The use of single‐species management tactics could also benefit from this broader perspective. Strategies might include considering how generalist consumers shape spatial food web structures when planning for species redistribution, whether through managing habitat for connectivity to facilitate animal dispersal or human‐facilitated translocations to enhance resilience in vulnerable populations. By embracing this perspective on asymmetric rewiring, we can better safeguard the resilience and functions of ecosystems in a changing world.

## Author Contributions

C.A.W. led the investigation and writing supervised by K.S.M. and T.D.T. C.A.W., T.D.T., K.S.M. and I.D. developed the methodology. Visualisation was conducted by C.A.W., C.B. and K.R.S.H. All authors contributed to conceptualisation and revisions.

## Peer Review

The peer review history for this article is available at https://www.webofscience.com/api/gateway/wos/peer‐review/10.1111/ele.70174.

## Supporting information


Appendix S1.

## Data Availability

All code to reproduce the theoretical simulations (Julia) presented in the main text and Appendix S1, as well as the full set of studies included in the habitat coupling review (Dataset S1), are available in Zenodo at https://doi.org/10.5281/zenodo.15706864.
