# Supplementary material for: Global Change Asymmetrically Rewires Ecosystems
Source: Ecol Lett. 2025 Jul 10;28(7):e70174. doi: 10.1111/ele.70174 (PMC12243702; doi:10.1111/ele.70174)
Supplement: Supplementary file 1 — Appendix S1. [file ELE-28-0-s001.docx]

**Appendix S1:** Global Change Asymmetrically Rewires Ecosystems

Charlotte A. Ward, Tyler D. Tunney, Ian Donohue, Carling Bieg, Kayla R. S. Hale, Bailey C. McMeans, John C. Moore, Kevin S. McCann

Corresponding Author: Charlotte A. Ward

Email: cward@uoguelph.ca

**This file includes:**

Supporting text

Figures S1 to S3

Table S1

SI References

**Data Availability Statement:**

All code to reproduce the theoretical simulations (Julia) presented in the main text and Appendix S1, as well as the full set of studies included in the habitat coupling review (Dataset S1), are available in Zenodo at <https://doi.org/10.5281/zenodo.15706864>.

**Review of the habitat coupling literature**

Our literature review aimed to identify studies that explore the effects of anthropogenic pressures on a generalist consumers’ habitat coupling using stable isotope analysis, energetic models, or gut content analysis. We employed a vote count approach to categorize studies that met our criteria and analyze whether food webs are being asymmetrically rewired by various anthropogenic pressures. The pressures examined include climate variability and change, land conversion, biological invasions, nutrient application to agricultural systems and other forms of pollution (nutrient application is considered a form of pollution in our analysis), as they have been identified as direct drivers of ecosystem change globally (Millennium Ecosystem Assessment, 2005; Nelson et al., 2006). Additionally, we included exploitation (Estes et al., 2011; Pauly et al., 1998), aquaculture (Gutgesell et al., 2022), and water diversion (de Guzman et al., 2022; Holmquist & Waddle, 2013) as anthropogenic pressures in our analysis, as they have also been identified as important drivers of global change in the ecological literature. We focused on upper trophic level species (secondary consumers and predators) because they are expected to reflect the asymmetric impacts of global change on distinct habitats most strongly (McCann, 2007; McCann et al., 2005; Rooney et al., 2008). To conduct our review of the habitat coupling literature, we performed searches using two major literature databases, PubMed, and Web of Science (WoS). We conducted an additional search using the same terms in Google Scholar to capture any remaining studies that may not have been indexed by PubMed or Web of Science. This approach ensured that articles unavailable in one database might be captured by the others.

*Search Algorithm*

The PubMed and WoS databases were queried by restricting the searches to the abstract and title fields to ensure a focused retrieval of relevant literature. Reviews were excluded to focus on primary research articles only. The Boolean search strings used combined terms related to diet shifts, habitat coupling, and food web structure with various global change drivers. The complete search algorithms are listed below:

((habitat coupling) OR (cross-ecosystem subsidies) OR (cross-ecosystem subsidy) OR (habitat compartment) OR (resource compartment) OR (resource subsidy) OR (resource subsidies) OR (food web architecture) OR (biostructure) OR (food web contraction) OR (food web expansion) OR (food web structure) OR (food web structural shift) OR (diet shift)) AND ((anthropogenic) OR (global change) OR (climate change) OR (warming) OR (anthropogenic stressor) OR (pollution) OR (harvest) OR (harvesting) OR (agriculture) OR (agricultural) OR (eutrophication) OR (brownification) OR (nutrient loading) OR (land-use) OR (land use) OR (land conversion) OR (species invasion) OR (invasive species) OR (species introduction) OR (biological invasion) OR (benthification) OR (exploitation) OR (poaching) OR (deforestation) OR (dam) OR (water diversion) OR (drought)) AND ((isotope) OR (stable isotope) OR (carbon flow) OR (energy flow) OR (energy flux) OR (energy pathway) OR (gut content) OR (stomach content) OR (LIM) OR (flexweb) OR (linear inverse) OR (adapted food-web energetic) OR (mass-balance) OR (Ecosim) OR (Ecopath))

*Screening and Selection*

The combined searches produced a total of 2,102 primary research articles after removing duplicate titles (databases accessed on April 19^th^, 2025). To filter these results, we employed a multi-step screening process guided by two decision flow charts (Figure S1 and Figure S2). The preliminary abstract screening (Figure S1) aimed to exclude obviously irrelevant papers and focus on those that addressed consumer habitat coupling or diet shifts across a spatial, temporal, or spatiotemporal gradient of anthropogenic pressures associated with global change.

Following the initial screening, we conducted a detailed assessment of the remaining papers using the workflow shown in Figure S2. This assessment involved several key steps. First, we identified the specific organisms or communities along with the ecosystem types examined in each study. We also recorded whether the studies explored temporal, spatial, or spatiotemporal gradients of anthropogenic pressures and categorized them into specific anthropogenic pressures, such as climate change, pollution, or biological invasions (Figure S2).

We defined a critical inclusion criterion where studies had to examine the reliance on or intake of energy derived from distinct habitats by generalist consumers (i.e. secondary consumers and predators. Studies that met this criterion fell into three methodological categories: stable isotope analysis, gut content analysis, and energetic modelling.

*Stable Isotope Analysis*

Studies employing isotope analysis (isotopes including: δ^13^C, δ^15^N, δ^34^S, δ^2^H, δ^87^Sr, δ^18^O; Zimmo et al., 2012) were identified and included in our final dataset if they used mixing models (e.g., two-source mixing models; Karlsson & Byström, 2005; Post, 2002; Vander Zanden et al., 1999; Bayesian mixing models; Parnell et al., 2010; Phillips et al., 2014; Stock et al., 2018) with baseline organisms rather than relying on raw stable isotope values, as baseline values of δ^13^C, δ^15^N, δ^34^S, δ^2^H, δ^87^Sr, and δ^18^O are known to vary across space (Fry, 2006; Zimmo et al., 2012). The outputs of these models were then used to assess whether generalist consumers increased habitat coupling (i.e., showed more even assimilation of resources from spatially distinct resources), decreased their habitat coupling (i.e., relied more heavily on a single resource), or exhibited no change across a gradient of spatial, temporal, or spatiotemporal anthropogenic pressures, or an experimental gradient of simulated anthropogenic pressure. If a study did not use mixing models with baseline organisms but did corroborate their results based on raw isotope values with an additional technique, such as gut content analysis or fatty acid analysis, we considered it a robust analysis of changes in the dietary reliance of a consumer on spatially distinct energy pathways and thus included the study in our final dataset (Dataset S1). Note that any studies using stable isotopes and gut content analysis were categorized under the methodological approach of “stable isotope analysis”, whereas studies only using gut content analysis were categorized under the methodological approach of “gut content analysis”. We excluded studies that used metrics of niche width, such as Layman’s metrics (Layman et al., 2007), without characterizing a consumer’s use of resources from specific habitats. Changes in niche width do not necessarily indicate a change in the reliance of a consumer species on different energy pathways and may instead reflect other aspects of foraging behaviour, such as feeding on novel resources within the same energy pathway or altering their reliance on species of different trophic levels. These criteria reduced the number of articles to a final list of 64 studies (Dataset S1).

*Gut Content Analysis*

We retained studies that used gut content analysis (also referred to as stomach content analysis) to quantify a consumer’s short-term reliance on resources from different habitats. To meet inclusion criteria, prey items had to be categorized by habitat of origin, and the study had to report either the proportion of total gut content weight or the relative frequency of prey types across habitats in consumer gut contents (Colborne et al., 2015; Wallace et al., 1997). Studies were excluded if they did not relate prey type to a specific habitat compartment or if they lacked a clear spatial component in interpreting dietary shifts. We relied on the author’s assertions regarding the spatial distinctness of prey sources and did a cursory check of each study using gut content analysis to ensure these assertions met our definition of spatially distinct energy pathways (Table 1 in main text). To determine whether habitat coupling increased, decreased, or did not change, we evaluated whether the proportion or frequency of prey items in the consumer’s gut contents shifted toward a more even reliance on prey from multiple habitat types (i.e., increased coupling), toward dominance by prey from a single habitat (i.e., decreased coupling), or showed no change across a spatial or temporal gradient of anthropogenic pressure.This yielded an additional 9 studies that were included in our final dataset (Dataset S1).

*Energetic Modelling*

Finally, we included studies that estimated the flow of energy from spatially distinct resource pools to generalist consumers using modelling approaches such as linear inverse modelling (van Oevelen et al., 2010), Ecopath with Ecosim (Christensen & Walters, 2004), or food-web energetics models (Gauzens et al., 2019; Jochum et al., 2021; Moore & de Ruiter, 2012). To be included in the final dataset (Dataset S1), these studies had to present spatially explicit estimates of energy or biomass transfer through the food web. To calculate flows, authors had to make use of biomass data and explicitly state the spatial and temporal scale at which biomass data was collected. In addition, in cases where this was a component of the modelling approach, the authors must have reported the sources used to make assumptions about dietary preference, metabolic scaling, and assimilation efficiencies (e.g., Potapov et al., 2024). To evaluate changes in habitat coupling, we examined whether the proportion of energy flow to generalist consumers from spatially discrete resource pools, which is often represented by functional groups, became more evenly distributed (increased coupling), more concentrated on a single energy pathway (decreased coupling), or remained unchanged across gradients of anthropogenic pressure. We excluded studies that used these approaches without a spatial dimension or without identifying consumer-level changes in energy intake (i.e., only energy flows among lower trophic level organisms were quantified). In total, 12 studies employing energetic modelling approaches met these criteria and were included in Dataset S1.

*Vote Count of Study Results*

We relied on the author’s findings reported in the results and discussion sections of each article to determine the directional change in habitat coupling by a generalist consumer. We categorized these changes as an increase, decrease, or no change in habitat coupling and recorded this information in our literature table (Dataset S1). We documented where this result appears in each study in our literature table, as well as the methodological approach used by the authors to form their conclusions. A summary of the directional results across different anthropogenic pressures are shown in Table S1. Studies that found an increase or decrease in habitat coupling (i.e., detected a change in a consumer’s dietary reliance on different food web compartments) were considered to show an asymmetric rewiring response to anthropogenic pressure.

For studies that detected asymmetric rewiring, we also identified the underlying mechanism driving the increase or decrease in consumer habitat coupling. This was determined by examining the articles to identify whether the authors reported or inferred changes in the accessibility of habitats used by a consumer, such as one habitat becoming physiologically stressful due to environmental conditions (Tunney et al., 2014) or other barriers preventing the consumer from accessing resources within that habitat (Bartels et al., 2016), or if they reported or inferred changes in the density of resources in different habitats used by consumers due to anthropogenic pressures (Champagne et al., 2022).

**Figure S1.** *Abstract and title decision flow chart*. Initial criteria used to identify studies that explore the effects of anthropogenic pressures associated with global change on the habitat coupling of generalist consumers. This process was based solely on the abstract and title of each study. When uncertain about whether a study should be included from the abstract and title, the row in our literature table containing the study information was highlighted in red to indicate further evaluation is required using the workflow in Figure S2.

**Figure S2.** *Full-article decision flow chart*. The process used to eliminate studies that did not explicitly evaluate generalist consumer habitat coupling across a temporal, spatial, or spatiotemporal gradient of anthropogenic pressure, or in response to experimentally driven changes simulating anthropogenic pressure. Following this detailed assessment, 85 studies were included in the final list of primary research articles. For each included study, information regarding the organisms, ecosystem, and the anthropogenic pressure explored was extracted. This process was ultimately used to identify the directional response of habitat coupling by predator species, provided the study met our criteria for inclusion.

**Table S1.** Summary of empirical findings from studies included in the literature review that assessed changes in generalist consumer habitat coupling in response to anthropogenic pressures. Studies are organized by system type (aquatic or terrestrial), ecosystem, and category of anthropogenic pressure, progressing from broader to more specific groupings. Each row indicates whether the study results show an increase, decrease, or no change in habitat coupling. Citations for each study are listed in the rightmost column and correspond to the full references at the end of this document.

| **Type** | **Ecosystem** | **Anthropogenic pressure** | **Δ Habitat coupling** | **References** |
| --- | --- | --- | --- | --- |
| Aquatic | Estuary | Biological invasion | Decrease | Li et al., 2023 |
|  |  |  | Increase | Laffaille et al., 2005 |
|  |  | Exploitation | Increase | Drago et al., 2018 |
|  |  | Multi-pressure | Decrease | Lafage et al., 2021 |
|  |  | Pollution | Decrease | Yogi et al., 2024 |
|  | Lake | Biological invasion | Decrease | Jaeger Miehls et al., 2009; Lesser et al., 2024; Vander Zanden et al., 1999; Eagles-Smith et al., 2008; Turschak et al., 2014 |
|  |  |  | Increase | Colborne et al., 2015; Hares et al., 2015; Rennie et al., 2009; Ozersky et al., 2012; Prestie et al., 2019 |
|  |  |  | No change | Barst et al., 2020 |
|  |  | Climate change | Decrease | Tunney et al., 2014; Bartels et al., 2016 |
|  |  |  | Increase | Hayden et al., 2015; Guzzo et al., 2017; Tunney et al., 2018; de Necker et al., 2022 |
|  |  | Land conversion | Decrease | Keva et al., 2025 |
|  |  |  | Increase | Glaz et al., 2014 |
|  |  | Multi-pressure | Decrease | Stone et al., 2020; Hayden et al., 2019 |
|  |  | Pollution | Decrease | Ripku et al., 2023 |
|  |  |  | Increase | Xu et al., 2014 |
|  |  |  | No change | Saboret et al., 2023 |
|  |  | Water diversion | Decrease | Mao et al., 2021 |
|  | Marine | Aquaculture | Increase | Sardenne et al., 2020 |
|  |  | Climate change | Decrease | Ullah et al., 2018; van den Berg et al., 2020; Braeckman et al., 2020 |
|  |  |  | Increase | Vernet et al., 2017; Gomes et al., 2024; Yurkowski et al., 2020; Ulrich and Tallman, 2021; Carlyle et al., 2022 |
|  |  | Exploitation | Increase | Gómez-Campos et al., 2011 |
|  |  | Land conversion | No change | Viana et al., 2015 |
|  |  | Multi-pressure | Decrease | Hempson et al., 2017; Rayner et al., 2021 |
|  |  |  | Increase | Jory et al., 2021 |
|  | Pond | Biological invasion | Decrease | Gobel et al., 2023 |
|  |  | Land conversion | Increase | Doi et al., 2010 |
|  | River | Biological invasion | Decrease | Wood et al., 2017 |
|  |  |  | No change | Dunlop et al., 2020 |
|  |  | Multi-pressure | Increase | Scharnweber et al., 2024 |
|  |  | Pollution | Decrease | East et al., 2017 |
|  |  | Water diversion | Decrease | Roussel et al., 2024 |
|  |  |  | Increase | Čivas et al., 2016 |
|  |  |  | No change | Aguiar-Santos et al., 2022 |
|  | Stream | Biological invasion | No change | Roon et al., 2016 |
|  |  | Climate change | Decrease | Jackson et al., 2024 |
|  |  | Land conversion | Decrease | England and Rosemond, 2004; Champagne et al., 2022; Effert-Fanta et al., 2022; Marker et al., 2023 |
|  |  | Multi-pressure | Increase | de Guzman et al., 2024 |
|  |  | Pollution | Increase | de Guzman et al., 2023; Kraus et al., 2015 |
|  |  |  | No change | Medinski et al., 2022 |
|  |  | Water diversion | Decrease | Cross et al., 2013 |
|  | Wetland | Pollution | No change | Sierszen et al., 2006 |
| Terrestrial | Forest | Climate change | Decrease | Manlick et al., 2024 |
|  |  | Land conversion | Decrease | Wang et al., 2024 |
|  |  | Land conversion | Increase | Potapov et al., 2024 |
|  |  | Multi-pressure | Decrease | Schwarz et al., 2017 |
|  | Riparian | Pollution | No change | Huszarik et al., 2024 |
|  |  |  | Increase | Sullivan et al., 2018 |
|  |  |  | Decrease | Hunt et al., 2020 |
|  |  | Climate change | No change | Gutiérrez-Fonseca et al., 2023 |
|  |  | Land conversion | Decrease | Stenroth et al., 2015 |
|  |  | Water diversion | No change | Sullivan et al., 2018 |
|  | Tundra | Climate change | Decrease | Almela et al., 2023 |
|  |  |  | No change | Carbonell Ellgutter et al., 2020 |
|  |  |  | Increase | McKinney et al., 2013 |
|  | Grassland | Multi-pressure | No change | Fox-Dobbs et al., 2012 |
|  | Island | Land conversion | Decrease | Matsubayashi et al., 2015 |
|  | Shrubland | Land conversion | Decrease | Larson et al., 2020 |

**Theoretical simulations**

In our theoretical example (Figure 4i-vi in main text), we use a standard five-species generalist food web module (McCann et al., 2005) to elucidate how the differential impacts of anthropogenic pressures on distinct habitats may rewire (restructure) food webs and alter the resilience and functions of ecosystems. The model consists of two basal resources, where R_1_ (Eqn. 1) and R_2_ (Eqn. 3) occupy Habitat 1 and 2 respectively, have logistic population growth, and do not interact with one another. Further, C_1_ (Eqn. 2) and C_2_ (Eqn. 4) represent two distinct consumer populations in which C_1_ forages exclusively on R_1_, while C_2_ forages exclusively on R_2_. The top predator in our model, P (Eqn. 5), is a generalist consumer species that forages on both C_1_ and C_2_ but is not omnivorous and therefore does not consume R_1_ or R_2_. The predator prefers to consume prey (C_1_ or C_2_) from the habitat that has greatest resource availability. Specifically, we incorporate a density-dependent behavioral response of the predator to changes in the densities of C_1_ and C_2_. All simulations were implemented in the Julia programming language (version 1.11.2)

*Model* *Equations*

We use the following system of equations to represent our food web:

$\frac{{dR}_{1}}{dt}=r_{R1}R_{1}\left( 1- \frac{R_{1}}{K_{1}} \right)-\frac{a_{R1C1}C_{R1C1}R_{1}}{\left( 1+ a_{R1C1}h_{R1C1}R_{1} \right)}$ (1)

$\frac{{dC}_{1}}{dt}=\frac{e_{R1C1}a_{R1C1}C_{1}R_{1}}{(1 + a_{R1C1}h_{R1C1}R_{1})}-{\frac{\Omega a_{C1P}PC_{1}}{\left( 1 + \Omega a_{C1P}h_{C1P}C_{1}+\left( 1-\Omega\right)a_{C2P}h_{C2P}C_{2} \right)}-m}_{C1}C_{1}$ (2)

$\frac{{dR}_{2}}{dt}=r_{R2}R_{2}\left( 1- \frac{R_{2}}{K_{2}} \right)-\frac{a_{C2R2}C_{2}R_{2}}{\left( 1 + a_{C2R2}h_{C2R2}R_{C2R2} \right)}$(3)

$\frac{{dC}_{2}}{dt}=\frac{e_{R2C2}a_{R2C2}C_{2}R_{2}}{(1 + a_{R2C2}h_{R2C2}R_{R2C2})}-{\frac{\left( 1-\Omega\right)a_{C2P}PC_{2}}{\left( 1 + \Omega a_{C1P}h_{C1P}C_{1}+\left( 1-\Omega\right)a_{C2P}h_{C2P}C_{2} \right)}-m}_{C2}C_{2}$ (4)

$\frac{dP}{dt}=\frac{e_{C1P}\Omega a_{C1P}PC_{1}{+ e}_{C2P}\left( 1-\Omega\right)a_{C2P}PC_{2}}{\left( 1 + \Omega a_{C1P}h_{C1P}C_{1}+\left( 1-\Omega\right)a_{C2P}h_{C2P}C_{2} \right)}-m_{p}P$(5)

Here, *r_i_* represents the intrinsic rate of population growth for the resource species *i* (R_1_ or R_2_), *K_i_* is the resource species *i*’s carrying capacity (also a surrogate for habitat productivity), *a_ij_* represents the maximum attack rate of species *j* on species *i*, *h_ij_* is the handling time for species *j* when consuming species *i*, *e_ij_* is the energy conversion efficiency of species *j* consuming species *i,* and *m_i_* is the natural mortality rate for species *i.*

With this model, the predator’s functional response (F) to C_1_ follows the form:

$F_{C1=}\frac{\Omega a_{C1P}C_{1}}{1+\Omega a_{C1P}h_{C1P}C_{1}+\left( 1-\Omega\right)a_{C2P}h_{C2P}C_{2}}$ (6)

While the predator’s functional response (FR) to C_2_ follows the form:

$F_{C2=}\frac{(1-\Omega)a_{C2P}C_{2}}{1+\Omega a_{C1P}h_{C1P}C_{1}+\left( 1-\Omega\right)a_{C2P}h_{C2P}C_{2}}$ (7)

Here, Ω measures the predator’s (P) preference scaling of its attack rate on C_1_ versus C_2_. Preference (Ω) is density-dependent, and the predator’s behavioral preference is modelled as:

$\Omega=\frac{\omega C_{1}}{\omega C_{1}+\left( 1-\omega\right)C_{2}}$ (8)

where *ω* is a scaling parameter that determines how the predator’s attack rates on C_1_ and C_2_ respond to changes in their availability. Specifically, *ω* represents the inherent preference for C_1_, with values of *ω* above 0.5 indicating a stronger preference for C_1_ and values below 0.5 indicating a stronger preference for C_2_. When *ω =* 0.5, the preference for C_1_ or C_2_ is entirely dependent on the density of the consumer populations. This equation allows for a non-linear functional response, where the predator's preference dynamically adjusts based on the current densities of C_1_ and C_2_. In our theoretical example, we set *ω =* 0.6 to simulate a food web in which the predator has a very slight inherent preference for C_1_ versus C_2_. We include this slight preference for C_1_ so that the initial conditions of the system produce a modestly asymmetric food web structure such that the trophic interactions among the predator and C_1_ are marginally stronger than those between the predator and C_2_. Asymmetries in food webs are ubiquitous in nature (Rooney et al., 2006) and therefore included here, however, the results are qualitatively the same whether the inherent preference (i.e., *ω*) of the predator is split evenly between both consumers and if there is a slight preference for C_1_.

*Theoretical Experiment: Simulating Differential Impacts of Anthropogenic Pressure on Habitat Productivity*

In our experiment, we vary *K_1_* while keeping *K_2_* constant to study the influence of differential changes in habitat productivity (i.e., resource carrying capacity) on food web dynamics. Here, changes in habitat productivity are analogous to alterations to physical habitats brought on by anthropogenic pressures, such as habitat degradation from land conversion. This may reflect changes in the availability of resources via altered habitat accessibility or resource density.

We track changes in response variables associated with food web structure (predator’s degree of habitat coupling; predator:consumer biomass ratios in Habitat 1), ecosystem functions (primary production and secondary (predator) production), and resilience (maximum real eigenvalues and predator population CV), as we decrease *K_1_* from a value of 1.395 to 0.76. We also track changes in the energy available to the predator (i.e., the combined consumer C_1_ and C_2_ production; Figure S3a), as well as the predator’s equilibrium density across this gradient (Figure S3b). The gradient used in our experiment encompasses a highly productive energy pathway in Habitat 1 when *K_1_* is high, to such low productivity conditions that C_1_ cannot be supported by the basal resources in Habitat 1 and experiences population collapse when *K_1_* is low.

In this experiment, parameters other than *K_1_* are held constant (other parameter values listed at the end of this section). All simulations were run for 2000-time steps. For all calculations, except for the maximum real eigenvalues (used in local stability analysis; see below for when equilibrium was calculated), we averaged the density of each state variable over time steps 1000 to 2000, thus removing the effect of transient dynamics. This averaging ensures that the values of each measure represent the system's behavior once it has reached an asymptotic state (i.e., stable equilibrium or stable limit cycle). Note that the resilience responses shown in Figure 4b. v and Figure 4b. vi reflect a well-studied pattern (McCann et al., 2005; Rooney et al., 2006), where intermediate levels of habitat coupling beget the greatest dynamical stability. However, by adjusting the parameters such that the system remains consistently low in productivity or very high in productivity across the gradient of *K_1_*, the system can be purely stabilized (left side of the hump in Figure 4b. v) or destabilized (right side of the hump in Figure 4b. v) by increasing the predator’s degree of habitat coupling. In other words, there are specific conditions under which the stability response exhibits only one side of the expected stability pattern.

*Measures of Food Web Structure*

To assess how food web structure responds to differential changes in habitat productivity, we quantified habitat coupling by the predator (P) as the evenness of its feeding across the two consumer trophospecies (C_1_ and C_2_). This was calculated using the predator’s functional responses (*F_C1_* [Eqn. 6] and *F_C2_* [Eqn. 7]*​*) following Eqn. 9. A value of 0.5 indicates perfectly even feeding across both consumers (i.e., strong habitat coupling), while values closer to 0 indicate increasing dominance of one consumer in the predator’s diet.

$Habitat coupling=0.5-|0.5-\frac{F_{C1}}{F_{C1}+ F_{C2}} |$ (9)

Further, we calculated the predator to consumer biomass ratios in Habitat 1 (P:C_1_) and 2 (P:C_2_) when the system reaches an asymptotic state to determine the top-heaviness of each food web compartment across the gradient of *K_1_.* This was calculated by dividing the predator’s mean density by the consumer’s mean density (C_1_). We use the term biomass ratio here because these values can be interpreted as either density or biomass, but biomass is the more common empirical unit for such ratios in biological systems.

*Measures of Ecosystem Function*

Next, we calculated measures associated with production (i.e., a key function of ecosystems) across the gradient of *K_1_* to gain insight into the impact of asymmetric changes in habitat productivity on energy flow through food webs. Primary production was calculated as the realized growth of basal resources R_1_ and R_2_ (at equilibrium) following Eqn.10. Secondary production was calculated as the total production of the predator population (at equilibrium) from consuming C_1_ and C_2_ following Eqn. 11.

$Primary production=r_{1}R_{1}\left( 1- \frac{R_{1}}{K_{1}} \right)+ r_{2}R_{2}(1- \frac{R_{2}}{K_{2}})$ (10)

$Secondary production=\frac{\Omega a_{C1P}C_{1}e_{C1P}P+ \left( \Omega-1 \right)a_{C2P}C_{2}e_{C2P}P}{1+\Omega a_{C1P}h_{C1P}C_{1}+\left( 1-\Omega\right)a_{C2P}h_{C2P}C_{2}}$ (11)

*Measures of Resilience*

To measure resilience, we performed local stability analyses by calculating the Jacobian matrix of the system at equilibrium and extracting its eigenvalues across the gradient of *K_1_*. The real part of the maximum eigenvalue ($\lambda$) was used to determine local stability (May, 1971; Pimm, 1979, 1980), with a negative value indicating stable dynamics and a positive value indicating unstable dynamics. This analysis allowed us to examine the stability of the system’s equilibrium across a gradient of asymmetric changes in habitat productivity (i.e., varying *K_1_*). It is important to note that altering the values of the other parameters in our model can produce dynamics that are non-excitable across the range of *K_1_* values used in our experiment. In such conditions, the destabilizing effect of increased habitat coupling may not be observed when *K_1_* is high. Nevertheless, the results are qualitatively consistent such that local stability follows a unimodal response to increasing habitat coupling across a gradient of a very low productivity system (i.e., habitat coupling is typically stabilizing) to very high productivity (i.e., habitat coupling is typically destabilizing).

To simulate environmental variability, stochasticity was introduced into the model by adding Gaussian white noise to each of the state variables at every time step. The noise had a magnitude (standard deviation) of 0.003, representing small random fluctuations around the state variable values at each time step. The noise was applied independently to each state variable, meaning that at each time step, the environmental noise affecting one population was not correlated with the noise affecting another.

We conducted the stochastic simulations and calculated the coefficient of variation (CV) of the predator population to assess the impact of asymmetric changes in habitat productivity on the resilience of the predator population in a variable world. Predator CV was calculated by dividing the standard deviation of the predator’s population density across 1000-time steps by the mean of the predator’s population density across this same time interval (Eqn. 12). More resilient predator populations were indicated by lower CV, while higher values of CV indicated less resilient predator populations.

$CV=\frac{\sigma}{\mu}$ (12)

**
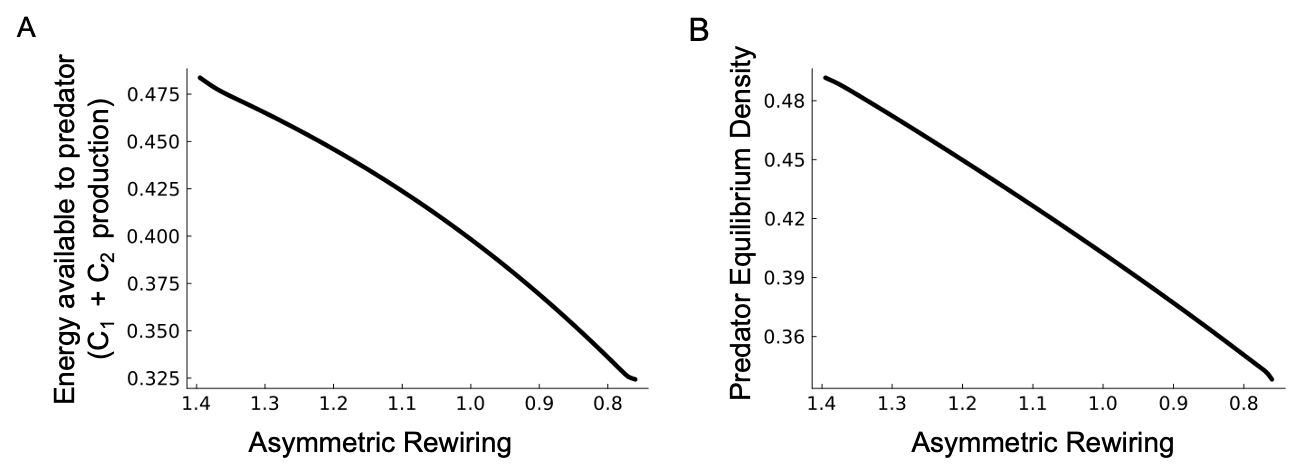
**

**Figure S3**. Consequences of differential changes in Habitat 1 and Habitat 2’s productivity on predator energy availability and equilibrium density. **(A)** Total energy available to the predator, calculated as the combined production of its two prey species (C₁ and C₂), declines with decreasing productivity in Habitat 1 (*K₁*). This reduction reflects the collapse of the C₁ population due to limited basal resources (R₁), while C₂ remains relatively unaffected. **(B)** The predator’s equilibrium population density decreases in response to reduced prey availability, highlighting the risk of predator decline or extinction as differential impacts on habitat intensify.

*Values of parameters held constant throughout the simulations*

*R_1_ parameters:
r_1_ = 2.0*

*R_2_ parameters:
r_2_ = 2.0
K_2_ = 2.0

C_1_ parameters:*

*a_R1C1_ = 0.9
h_R1C1_ = 0.6
e_R1C1_ = 0.7
m_C2_ = 0.3*

*C_2_ parameters:
a_R2C2_ = 0.9
h_R2C2_ = 0.6
e_R2C2_ = 0.7
m_C2_ = 0.3*

*P parameters:*

*a_C1P_ = 1.2
h_C1P_ = 0.6
e_C1P_ = 0.7*

*a_C2P_ = 1.2
h_C2P_ = 0.6
e_C2P_ = 0.7
m_P_ = 0.3
ω = 0.6*

**SI References**

Aguiar-Santos, J., deHart, P. A. P., Forsberg, B. R., and C. E. C. Freitas. 2022. “Isotopic niche alteration of a predator fish in a dammed Amazonian black water river.” *Journal of Fish Biology* **101**: 1530–1539.

Almela, P., Rico, E., Velázquez, D., Verleyen, E., and A. Quesada. 2023. “Soil moisture drives differences in the diversity and trophic complexity of high Arctic tundra soils.” *FEMS Microbiology Ecology* **99**: 1–11.

Barnes, A. D., Jochum, M., Lefcheck, J. S., Eisenhauer, N., Scherber, C., M. I. O’Connor, et al. 2018. “Energy flux: the link between multitrophic biodiversity and ecosystem functioning.” *Trends in Ecology & Evolution* **33**: 186–197.

Barst, B. D., Hudelson, K., Lescord, G. L., Santa-Rios, A., Basu, N., A. Crémazy, and P. E. Drevnick. 2020. “Effects of non-native fish on lacustrine food web structure and mercury biomagnification along a dissolved organic carbon gradient.” *Environmental Toxicology and Chemistry* **39**: 2196–2207.

Bartels, P., Hirsch, P. E., Svanbäck, R., and P. Eklöv. 2016. “Dissolved organic carbon reduces habitat coupling by top predators in lake ecosystems.” *Ecosystems* **19**: 955–967.

Braeckman, U., Soetaert, K., Pasotti, F., Quartino, M. L., Vanreusel, A., L. A. Saravia, et al. 2024. “Glacial melt impacts carbon flows in an Antarctic benthic food web.” *Frontiers in Marine Science* **11**: 1359597.

Carbonell Ellgutter, J. A., Ehrich, D., Killengreen, S. T., Ims, R. A., and E. R. Unnsteinsdóttir. 2020. “Dietary variation in Icelandic arctic fox (Vulpes lagopus) over a period of 30 years assessed through stable isotopes.” *Oecologia* **192**: 403–414.

Carlyle, C. G., Roth, J. D., Yurkowski, D. J., Kohlbach, D., Young, B. G., T. A. Brown, et al. 2022. “Spatial variation in carbon source use and trophic position of ringed seals across a latitudinal gradient of sea ice.” *Ecological Indicators* **145**: 109746.

Champagne, E. J., Guzzo, M. M., Gutgesell, M. K., and K. S. McCann. 2022. “Riparian buffers maintain aquatic trophic structure in agricultural landscapes.” *Biology Letters* **18**: 20210598.

Christensen, V., and C. J. Walters. 2004. “Ecopath with Ecosim: methods, capabilities and limitations.” *Ecological Modelling* **172**: 109–139.

Čivas, L., Kesminas, V., and R. Barisevičiūtė. 2016. “Impact of the riverbed morphology on the source of organic material and the trophic structure of fish community along the upper reaches of the Nevėžis River, Lithuania.” *Acta Ichthyologica et Piscatoria* **46**: 303–312.

Colborne, S. F., Clapp, A. D. M., Longstaffe, F. J., and B. D. Neff. 2015. “Foraging ecology of native pumpkinseed (Lepomis gibbosus) following the invasion of zebra mussels (Dreissena polymorpha).” *Canadian Journal of Fisheries and Aquatic Sciences* **72**: 983–990.

Cross, W. F., Baxter, C. V., Rosi-Marshall, E. J., Hall, R. O., Kennedy, T. A., K. C. Donner, et al. 2013. “Food-web dynamics in a large river discontinuum.” *Ecological Monographs* **83**: 311–337.

de Guzman, I., Elosegi, A., von Schiller, D., González, J. M., Paz, L. E., B. Gauzens, et al. 2023. “Treated and highly diluted, but wastewater still impacts diversity and energy fluxes of freshwater food webs.” *Journal of Environmental Management* **345**: 118510.

de Guzman, I., Montoya, J. M., Elosegi, A., Pérez-Calpe, A. V., von Schiller, D., J. M. González, and A. Larrañaga. 2024. “Food-web energy fluxes, energy transfer efficiency, and diversity respond distinctively to pollution and water diversion in rivers.” *Freshwater Biology* **69**: 351–364.

de Necker, L., Brendonck, L., Gerber, R., Lemmens, P., Soto, D. X., Y. Ikenaka, et al. 2022. “Drought altered trophic dynamics of an important natural saline lake: a stable isotope approach.” *Science of the Total Environment* **834**: 155338.

Doi, H., Chang, K. H., Ando, T., Imai, H., and S. Nakano. 2010. “Shoreline bank construction modifies benthic–pelagic coupling of food webs.” *Ecological Engineering* **36**: 601–604.

Drago, M., Franco-Trecu, V., Segura, A. M., Valdivia, M., González, E. M., A. Aguilar, and L. Cardona. 2018. “Mouth gape determines the response of marine top predators to long-term fishery-induced changes in food web structure.” *Scientific Reports* **8**: 1–12.

Dunlop, K., Eloranta, A. P., Schoen, E., Wipfli, M., Jensen, J. L. A., R. Muladal, and G. N. Christensen. 2021. “Evidence of energy and nutrient transfer from invasive pink salmon (Oncorhynchus gorbuscha) spawners to juvenile Atlantic salmon (Salmo salar) and brown trout (Salmo trutta) in northern Norway.” *Ecology of Freshwater Fish* **30**: 270–283.

Eagles-Smith, C. A., Suchanek, T. H., Colwell, A. E., Anderson, N. L., and P. B. Moyle. 2008. “Changes in fish diets and food web mercury bioaccumulation induced by an invasive planktivorous fish.” *Ecological Applications* **18**: A213–A226.

East, J. L., Wilcut, C., and A. A. Pease. 2017. “Aquatic food-web structure along a salinized dryland river.” *Freshwater Biology* **62**: 681–694.

Effert-Fanta, E. L., Fischer, R. U., and D. H. Wahl. 2022. “Riparian and watershed land use alters food web structure and shifts basal energy in agricultural streams.” *Aquatic Sciences* **84**: 1–18.

England, L. E., and A. D. Rosemond. 2004. “Small reductions in forest cover weaken terrestrial–aquatic linkages in headwater streams.” *Freshwater Biology* **49**: 721–734.

Estes, J. A., Terborgh, J., Brashares, J. S., Power, M. E., Berger, J., W. J. Bond, et al. 2011. “Trophic downgrading of planet Earth.” *Science* **333**: 301–306.

Fox-Dobbs, K., Nelson, A. A., Koch, P. L., and J. A. Leonard. 2012. “Faunal isotope records reveal trophic and nutrient dynamics in twentieth century Yellowstone grasslands.” *Biology Letters* **8**: 838–841.

Fry, B. 2006. *Stable isotope ecology*. New York: Springer.

Glaz, P., Sirois, P., Archambault, P., and C. Nozais. **2014**. “Impact of forest harvesting on trophic structure of eastern Canadian Boreal Shield lakes: insights from stable isotope analyses.” *PLOS ONE* 9: e96143.

Gobel, N., Laufer, G., González-Bergonzoni, I., Soutullo, Á., and M. Arim. **2023**. “Invariant and vulnerable food web components after bullfrog invasion.” *Biological Invasions* 25: 901–916.

Gomes, D. G. E., Ruzicka, J. J., Crozier, L. G., Huff, D. D., Brodeur, R. D., and J. D. Stewart. 2024. “Marine heatwaves disrupt ecosystem structure and function via altered food webs and energy flux.” *Nature Communications* **15**: 1–10.

Gómez-Campos, E., Borrell, A., Cardona, L., Forcada, J., and A. Aguilar. 2011. “Overfishing of small pelagic fishes increases trophic overlap between immature and mature striped dolphins in the Mediterranean Sea.” *PLOS ONE* **6**: e24554.

Gutgesell, M., McMeans, B. C., Guzzo, M. M., de Groot, V., Fisk, A. T., T. B. Johnson, and K. S. McCann. 2022. “Subsidy accessibility drives asymmetric food web responses.” *Ecology* **103**: e3817.

Gutiérrez-Fonseca, P. E., Pringle, C. M., Ramírez, A., Gómez, J. E., and P. García. 2024. “Hurricane disturbance drives trophic changes in neotropical mountain stream food webs.” *Ecology* **105**: e4202.

Guzzo, M. M., Blanchfield, P. J., and M. D. Rennie. 2017. “Behavioral responses to annual temperature variation alter the dominant energy pathway, growth, and condition of a cold-water predator.” *Proceedings of the National Academy of Sciences of the United States of America* **114**: 9912–9917.

Hares, C. J., Jonas, J. L., and J. B. K. Leonard. 2015. “Diet analysis of burbot (Lota lota) from eastern Lake Michigan: 1996–2012.” *Hydrobiologia* **757**: 89–99.

Hayden, B., Harrod, C., Thomas, S. M., Eloranta, A. P., Myllykangas, J. P., A. Siwertsson, et al. 2019. “From clear lakes to murky waters – tracing the functional response of high-latitude lake communities to concurrent ‘greening’ and ‘browning.’” *Ecology Letters* **22**: 807–816.

Hayden, B., Holopainen, T., Amundsen, P. A., Eloranta, A. P., Knudsen, R., K. Præbel, and K. K. Kahilainen. 2013. “Interactions between invading benthivorous fish and native whitefish in subarctic lakes.” *Freshwater Biology* **58**: 1234–1250.

Hempson, T. N., Graham, N. A. J., MacNeil, M. A., Williamson, D. H., Jones, G. P., and G. R. Almany. 2017. “Coral reef mesopredators switch prey, shortening food chains, in response to habitat degradation.” *Ecology and Evolution* **7**: 2626–2635.

Holmquist, J. G., and T. J. Waddle. 2013. “Predicted macroinvertebrate response to water diversion from a montane stream using two-dimensional hydrodynamic models and zero flow approximation.” *Ecological Indicators* **28**: 115–124.

Hunt, J. L., Paterson, H., Close, P., and N. E. Pettit. 2020. “Riparian condition influences spider community structure and the contribution of aquatic carbon subsidies to terrestrial habitats.” *Science of the Total Environment* **746**.

Huszarik, M., Roodt, A. P., Wernicke, T., Link, M., Lima-Fernandes, E., D. Åhlén, et al. 2024. “Shift in diet composition of a riparian predator along a stream pollution gradient.” *Proceedings of the Royal Society B: Biological Sciences* **291**: 20242104.

Jackson, M. C., O’Gorman, E. J., Gallo, B., Harpenslager, S. F., Randall, K., D. N. Harris, et al. 2024. “Warming reduces trophic diversity in high-latitude food webs.” *Global Change Biology* **30**: e17518.

Jochum, M., Barnes, A. D., Brose, U., Gauzens, B., Sünnemann, M., A. Amyntas, and N. Eisenhauer. 2021. “For flux’s sake: general considerations for energy-flux calculations in ecological communities.” *Ecology and Evolution* **11**: 12948–12969.

Jory, C., Lesage, V., Leclerc, A., Giard, J., Iverson, S., M. Bérubé, et al. 2021. “Individual and population dietary specialization decline in fin whales during a period of ecosystem shift.” *Scientific Reports* **11**: 1–14.

Karlsson, J., and P. Byström. 2005. “Littoral energy mobilization dominates energy supply for top consumers in subarctic lakes.” *Limnology and Oceanography* **50**: 538–543.

Keva, O., Cobain, M. R. D., Eloranta, A. P., Hämäläinen, H., Kiljunen, M., J. Schilder, and R. I. Jones. 2025. “The role of land use in terrestrial support of boreal lake food webs.” *Nature Communications* **16**: 1–12.

Kraus, J. M., Pomeranz, J. F., Todd, A. S., Walters, D. M., Schmidt, T. S., and R. B. Wanty. 2016. “Aquatic pollution increases use of terrestrial prey subsidies by stream fish.” *Journal of Applied Ecology* **53**: 44–53.

Lafage, D., Carpentier, A., Duhamel, S., Dupuy, C., Feunteun, E., S. Lesourd, and J. Pétillon. 2021. “Site characteristics more than vegetation type influence food web structure of intertidal salt marshes.” *Frontiers in Marine Science* **8**: 669759.

Laffaille, P., Pétillon, J., Parlier, E., Valéry, L., Ysnel, F., A. Radureau, et al. 2005. “Does the invasive plant Elymus athericus modify fish diet in tidal salt marshes?” *Estuarine, Coastal and Shelf Science* **65**: 739–746.

Larson, R. N., Brown, J. L., Karels, T., and S. P. D. Riley. 2020. “Effects of urbanization on resource use and individual specialization in coyotes (Canis latrans) in southern California.” *PLOS ONE* **15**: e0228881.

Layman, C. A., Arrington, D. A., Montaña, C. G., and D. M. Post. 2007. “Can stable isotope ratios provide for community-wide measures of trophic structure?” *Ecology* **88**: 42–48.

Lesser, J. S., Bruel, R., Marcy-Quay, B., McReynolds, A. T., Stockwell, J. D., and J. E. Marsden. 2024. “Whole-lake food web model indicates alewife invasion fueled lake trout restoration and altered patterns of trophic flow in Lake Champlain.” *Journal of Great Lakes Research* **50**: 102249.

Li, X., Yang, W., Ma, X., Zhu, Z., Sun, T., B. Cui, and Z. Yang. 2023. “Invasive Spartina alterniflora habitat forms high energy fluxes but low food web stability compared to adjacent native vegetated habitats.” *Journal of Environmental Management* **334**: 117487.

Magioli, M., Moreira, M. Z., Fonseca, R. C. B., Ribeiro, M. C., Rodrigues, M. G., and K. M. P. M. de B. Ferraz. 2019. “Human-modified landscapes alter mammal resource and habitat use and trophic structure.” *Proceedings of the National Academy of Sciences* **116**: 18466–18472.

Manlick, P. J., and J. N. Pauli. 2020. “Human disturbance increases trophic niche overlap in terrestrial carnivore communities.” *Proceedings of the National Academy of Sciences of the United States of America* **117**: 26842–26848.

Manlick, P. J., Perryman, N. L., Koltz, A. M., Cook, J. A., and S. D. Newsome. 2024. “Climate warming restructures food webs and carbon flow in high-latitude ecosystems.” *Nature Climate Change* **14**: 184–189.

Mao, Z., Gu, X., Cao, Y., Luo, J., Zeng, Q., H. Chen, and E. Jeppesen. 2021. “Pelagic energy flow supports the food web of a shallow lake following a dramatic regime shift driven by water level changes.” *Science of the Total Environment* **756**.

Marker, J., Bergman, E., Bowes, R. E., and D. Lafage. 2023. “Small stream predators rely heavily on terrestrial matter energy input in the fall, regardless of riparian buffer size.” *Food Webs* **36**: e00302.

Matsubayashi, J., Morimoto, J. O., Tayasu, I., Mano, T., Nakajima, M., O. Takahashi, et al. 2015. “Major decline in marine and terrestrial animal consumption by brown bears (Ursus arctos).” *Scientific Reports* **5**: 1–8.

May, R. M. 1971. “Stability in multispecies community models.” *Mathematical Biosciences* **12**: 59–79.

McCann, K. 2007. “Protecting biostructure.” *Nature* **446**: 29.

McCann, K. S., Rasmussen, J. B., and J. Umbanhowar. 2005. “The dynamics of spatially coupled food webs.” *Ecology Letters* **8**: 513–523.

McKinney, M. A., Iverson, S. J., Fisk, A. T., Sonne, C., Rigét, F. F., R. J. Letcher, et al. 2013. “Global change effects on the long-term feeding ecology and contaminant exposures of East Greenland polar bears.” *Global Change Biology* **19**: 2360–2372.

Medinski, N. A., Maitland, B. M., Jardine, T. D., Drake, D. A. R., and M. S. Poesch. 2022. “A catastrophic coal mine spill in the Athabasca River watershed induces isotopic niche shifts in stream biota including an endangered rainbow trout ecotype.” *Canadian Journal of Fisheries and Aquatic Sciences* **79**: 1321–1334.

Miehls, A. L. J., Mason, D. M., Frank, K. A., Krause, A. E., Peacor, S. D., and W. W. Taylor. 2009. “Invasive species impacts on ecosystem structure and function: a comparison of Oneida Lake, New York, USA, before and after zebra mussel invasion.” *Ecological Modelling* **220**: 3194–3209.

Millennium Ecosystem Assessment. 2005. *Ecosystems and human well-being: synthesis.* Washington, DC: Island Press.

Moore, J. C., and P. C. de Ruiter. 2012. *Energetic food webs: an analysis of real and model ecosystems.* Oxford: Oxford University Press.

Nelson, G. C., Bennett, E., Berhe, A. A., Cassman, K., DeFries, R., T. Dietz, et al. 2006. “Anthropogenic drivers on ecosystem change: an overview.” *Ecology and Society* **11**: 29.

Ozersky, T., Evans, D. O., and D. R. Barton. 2012. “Invasive mussels alter the littoral food web of a large lake: stable isotopes reveal drastic shifts in sources and flow of energy.” *PLOS ONE* **7**: e51249.

Parnell, A. C., Inger, R., Bearhop, S., and A. L. Jackson. 2010. “Source partitioning using stable isotopes: coping with too much variation.” *PLOS ONE* **5**: e9672.

Pauly, D., Christensen, V., Dalsgaard, J., Froese, R., and F. Torres. 1998. “Fishing down marine food webs.” *Science* **279**: 860–863.

Phillips, D. L., Inger, R., Bearhop, S., Jackson, A. L., Moore, J. W., A. C. Parnell, et al. 2014. “Best practices for use of stable isotope mixing models in food-web studies.” *Canadian Journal of Zoology* **92**: 823–835.

Pimm, S. L. 1980. “Properties of food webs.” *Ecology* **61**: 219–225.

Post, D. M. 2002. “Using stable isotopes to estimate trophic position: models, methods, and assumptions.” *Ecology* **83**: 703–718.

Potapov, A. M., Drescher, J., Darras, K., Wenzel, A., Janotta, N., R. Nazarreta, et al. 2024. “Rainforest transformation reallocates energy from green to brown food webs.” *Nature* **627**: 116–122.

Rayner, M. J., Dunphy, B. J., Lukies, K., Adams, N. J., Berg, M., L. Kozmian-Ledward, et al. 2021. “Stable isotope record from a resident New Zealand seabird community suggests changes in distribution but not trophic position since 1878.” *Marine Ecology Progress Series* **678**: 171–182.

Rennie, M. D., Sprules, W. G., and T. B. Johnson. 2009. “Resource switching in fish following a major food web disruption.” *Oecologia* **159**: 789–802.

Riedl, H. L., Stinson, L., Pejchar, L., and W. H. Clements. 2018. “An introduced plant affects aquatic-derived carbon in the diets of riparian birds.” *PLOS ONE* **13**: e0207389.

Ripku, T., Hayhurst, L., Metcalfe, C. D., and M. Rennie. 2023. “Isotopic-based evidence for reduced benthic contributions to fish after a whole-lake addition of nanosilver.” *Journal of Fish Biology*.

Roon, D. A., Wipfli, M. S., Wurtz, T. L., and A. L. Blanchard. 2016. “Invasive European bird cherry (Prunus padus) reduces terrestrial prey subsidies to urban Alaskan salmon streams.” *Canadian Journal of Fisheries and Aquatic Sciences* **73**: 1679–1690.

Rooney, N., McCann, K. S., and J. C. Moore. 2008. “A landscape theory for food web architecture.” *Ecology Letters* **11**: 867–881.

Rooney, N., McCann, K., Gellner, G., and J. C. Moore. 2006. “Structural asymmetry and the stability of diverse food webs.” *Nature* **442**: 265–269.

Roussel, J. M., Fraisse, S., Dézerald, O., Fovet, O., Pannard, A., H. Rodriguez-Perez, et al. 2023. “Effects of large dams on the aquatic food web along a coastal stream with high sediment loads.” *Frontiers in Ecology and Evolution* **11**: 1250892.

Saboret, G., Stalder, D., Matthews, B., Brodersen, J., and C. J. Schubert. 2023. “Autochthonous production sustains food webs in large perialpine lakes, independent of trophic status: evidence from amino acid stable isotopes.” *Freshwater Biology* **68**: 870–887.

Scharnweber, K., Scholz, C., Schippenbeil, V., Milano, S., and D. Hühn. 2024. “Effects of mining activities on fish communities and food web dynamics in a lowland river.” *Ecology and Evolution* **14**: e11111.

Schwarz, B., Barnes, A. D., Thakur, M. P., Brose, U., Ciobanu, M., P. B. Reich, et al. 2017. “Warming alters energetic structure and function but not resilience of soil food webs.” *Nature Climate Change* **7**: 895–900.

Sierszen, M. E., Peterson, G. S., Trebitz, A. S., Brazner, J. C., and C. W. West. 2006. “Hydrology and nutrient effects on food-web structure in ten Lake Superior coastal wetlands.” *Wetlands* **26**: 951–964.

Stenroth, K., Polvi, L. E., Fältström, E., and M. Jonsson. 2015. “Land-use effects on terrestrial consumers through changed size structure of aquatic insects.” *Freshwater Biology* **60**: 136–149.

Stock, B. C., Jackson, A. L., Ward, E. J., Parnell, A. C., Phillips, D. L., and B. X. Semmens. 2018. “Analyzing mixing systems using a new generation of Bayesian tracer mixing models.” *PeerJ* **6**: e5096.

Stone, J. P., Pangle, K. L., Pothoven, S. A., Vanderploeg, H. A., Brandt, S. B., T. O. Höök, et al. 2020. “Hypoxia’s impact on pelagic fish populations in Lake Erie: a tale of two planktivores.” *Canadian Journal of Fisheries and Aquatic Sciences* **77**: 1131–1148.

Sullivan, S. M. P., Hossler, K., and L. A. Meyer. 2019. “Artificial lighting at night alters aquatic–riparian invertebrate food webs.” *Ecological Applications* **29**: e01821.

Sullivan, S. M. P., Manning, D. W. P., and R. P. Davis. 2018. “Do the ecological impacts of dam removal extend across the aquatic–terrestrial boundary?” *Ecosphere* **9**: e02180.

Tunney, T. D., McCann, K. S., Jarvis, L., Lester, N. P., and B. J. Shuter. 2018. “Blinded by the light? Nearshore energy pathway coupling and relative predator biomass increase with reduced water transparency across lakes.” *Oecologia* **186**: 1031–1041.

Tunney, T. D., McCann, K. S., Lester, N. P., and B. J. Shuter. 2014. “Effects of differential habitat warming on complex communities.” *Proceedings of the National Academy of Sciences of the United States of America* **111**: 8077–8082.

Turschak, B. A., Bunnell, D., Czesny, S., Höök, T. O., Janssen, J., D. Warner, et al. 2014. “Nearshore energy subsidies support Lake Michigan fishes and invertebrates following major changes in food web structure.” *Ecology* **95**: 1243–1252.

Ullah, H., Nagelkerken, I., Goldenberg, S. U., and D. A. Fordham. 2018. “Climate change could drive marine food web collapse through altered trophic flows and cyanobacterial proliferation.” *PLOS Biology* **16**: e2003446.

Ulrich, K. L., and R. F. Tallman. 2021. “The capelin invasion: evidence for a trophic shift in Arctic char populations from the Cumberland Sound region, Nunavut, Canada.” *Arctic Science* **7**: 413–435.

van den Berg, G. L., Vermeulen, E., Valenzuela, L. O., Bérubé, M., A. Ganswindt, et al. 2021. “Decadal shift in foraging strategy of a migratory Southern Ocean predator.” *Global Change Biology* **27**: 1052–1067.

van Oevelen, D., van den Meersche, K., Meysman, F. J. R., Soetaert, K., Middelburg, J. J., and A. F. Vézina. 2010. “Quantifying food web flows using linear inverse models.” *Ecosystems* **13**: 32–45.

Vander Zanden, M. J., Casselman, J. M., and J. B. Rasmussen. 1999. “Stable isotope evidence for the food web consequences of species invasions in lakes.” *Nature* **401**: 464–467.

Vernet, M., Richardson, T. L., Metfies, K., Nöthig, E.-M., and I. Peeken. 2017. “Models of plankton community changes during a warm water anomaly in Arctic waters show altered trophic pathways with minimal changes in carbon export.” *Frontiers in Marine Science* **4**: 244259.

Viana, I. G., Valiela, I., Martinetto, P., Monteiro Pierce, R., and S. E. Fox. 2015. “Isotopic studies in Pacific Panama mangrove estuaries reveal lack of effect of watershed deforestation on food webs.” *Marine Environmental Research* **103**: 95–102.

Wallace, J. B., Eggert, S. L., Meyer, J. L., and J. R. Webster. 1997. “Multiple trophic levels of a forest stream linked to terrestrial litter inputs.” *Science* **277**: 102–104.

Wang, J., Liu, T., Zhao, J., Ning, C., Chen, S., Zhang, X., Liu, G., Kuzyakov, Y., and W. Yan. 2024. “Energy flows through nematode food webs depending on the soil carbon and nitrogen contents after forest conversion.” *Science of The Total Environment* 935: 173322.

Wang, S. W., Springer, A. M., Budge, S. M., Horstmann, L., Quakenbush, L. T., and M. J. Wooller. 2016. “Carbon sources and trophic relationships of ice seals during recent environmental shifts in the Bering Sea.” *Ecological Applications* **26**: 830–845.

Wood, K. A., Hayes, R. B., England, J., and J. Grey. 2017. “Invasive crayfish impacts on native fish diet and growth vary with fish life stage.” *Aquatic Sciences* **79**: 113–125.

Xu, J., Wen, Z., Ke, Z., Zhang, M., Zhang, M., Guo, N., Hansson, L. A., and P. Xie. 2014. “Contrasting energy pathways at the community level as a consequence of regime shifts.” *Oecologia* **175**: 231–241.

Yogi, D. S., Naik, A., Yadav, R., Desai, A., and M. Nanajkar. 2024. “‘Trophic switch’ by catfish community from predation to scavenging modulated by human food discard in an estuarine bay.” *Environmental Science and Pollution Research* **31**: 9183–9196.

Yurkowski, D. J., Brown, T. A., Blanchfield, P. J., and S. H. Ferguson. 2020. “Atlantic walrus signal latitudinal differences in the long-term decline of sea ice-derived carbon to benthic fauna in the Canadian Arctic.” *Proceedings of the Royal Society B: Biological Sciences* **287**: 20202126.

Zhou, Z., Krashevska, V., Widyastuti, R., Scheu, S., and A. Potapov. 2022. “Tropical land use alters functional diversity of soil food webs and leads to monopolization of the detrital energy channel.” *eLife* **11**.

Zimmo, S., Blanco, J., and S. Nebel. 2012. “The use of stable isotopes in the study of animal migration.” *Nature Education Knowledge* **3**: 3.
